# Supplementary material for: Systematic Assessment of Burst Impurity in Confocal-Based Single-Molecule Fluorescence Detection Using Brownian Motion Simulations
Source: Molecules. 2019 Jul 13;24(14):2557. doi: 10.3390/molecules24142557 (PMC6680824; doi:10.3390/molecules24142557)
Supplement: Supplementary file 1 [file molecules-24-02557-s001.zip › supplementary tables.pdf]

Table S1: Different 3D diffusion simulation conditions that were tested

**Box[1] x (μm)**	**Box y (μm)**	**Box z (μm)**	**Box vol.[2] (fL)**	**No. of molecules**	**Conc.[3] (pM)**	**D[4] (μm²/s)**	**Time[5] (s)**	**PSF model[6] (G/N)**
5.54	5.54	13.85	425	15	62	90.0	180	N
5.54	5.54	13.85	425	15	62	90.0	60	N
6.60	6.60	19.70	858	15	31	90.0	60	N
8.30	8.30	24.90	1715	15	15	90.0	60	N
5.54	5.54	13.85	425	15	62	22.5	60	N
5.54	5.54	13.85	425	15	62	5.6	180	N
5.54	5.54	13.85	425	15	62	5.6	60	N
5.54	5.54	13.85	425	15	62	90.0	180	G
5.54	5.54	13.85	425	15	62	90.0	60	G
6.60	6.60	19.70	858	15	31	90.0	60	G
8.30	8.30	24.90	1715	15	15	90.0	60	G
5.54	5.54	13.85	425	15	62	22.5	60	G
5.54	5.54	13.85	425	15	62	5.6	180	G
5.54	5.54	13.85	425	15	62	5.6	60	G

[1] The molecules diffuse inside a box having a length x, depth y & height z.

[2] The box volume is calculated from its dimensions

[3] The concentration is derived from the No. of molecules divided by Avogadro's number and then divided by the volume (in L units)

[4] The diffusion coefficient.

[5] The overall simulation time.

[6] The model of the point-spread-function (PSF) used in the simulation, either using the Gaussian approximation (G), or a numerically calculated PSF (N). See details in Materials and Methods.

Table S2: the values of all quantities assessed in this work for all simulations of single detection channel SMFD (not single-molecule FRET)

PSF[1] (G[2]/N[3])	C[4] (pM)	D[5] (μm²/s)	Sim time[6] (s)	BG rate[7] (Hz)	m[8]	F[9]	sz th[10]	w sz[11] (ms)	% photonic impurity[12]	Fraction of impure bursts[13] ($x10^{-3}$)	Mean burst impurity[14] ($x10^{-3}$)	Position dispersion – z[15] (nm)	Position dispersion – x[16] (nm)	Position dispersion – y[17] (nm)	FCS diffusion time[18] (ms)	FCS mean molecules in the PSF[19] ($x10^{-3}$)	P(N>1)[20] ($x10^{-3}$)	Mean burst width[21] (ms)
G	62	90	180	2433	5	6	5	0.0	2.2	67.9±5.3	13.8±0.7	555.1±0.9	118.8±0.2	118.3±0.2	2.8±1.9	17.1±1.2	8.5±0.6[22]	0.64±0.01
					10	6	10	0.0	2.8	112.1±8.9	20.2±1.1	535.0±0.9	118.7±0.2	118.0±0.2	2.9±2.0	15.5±1.1	7.7±0.5	1.26±0.02
					15	6	15	0.0	3.3	151.9±11.4	25.3±1.3	521.1±0.9	118.8±0.2	117.6±0.2	3.0±2.0	14.9±1.0	7.4±0.5	1.88±0.02
					20	6	20	0.0	3.8	180.7±13.5	30.1±1.6	510.8±0.9	118.7±0.2	117.5±0.2	3.1±2.1	14.4±1.0	7.2±0.5	2.52±0.03
					10	3	10	0.0	3.9	160.0±10.5	29.2±1.3	560.3±0.9	122.3±0.2	121.6±0.2	2.9±2.0	20.0±1.4	9.9±0.7	2.80±0.03
					10	11	10	0.0	2.1	86.9±7.5	15.6±0.9	503.6±0.9	114.0±0.2	113.1±0.2	2.8±1.9	12.6±0.9	6.3±0.4	0.67±0.01
					10	16	10	0.0	1.8	74.6±6.9	13.4±0.8	478.6±0.9	109.9±0.2	108.7±0.2	2.7±1.8	10.7±0.7	5.4±0.4	0.47±0.01
					10	21	10	0.0	1.8	62.8±6.4	11.6±0.8	457.5±0.9	105.7±0.2	104.6±0.2	2.5±1.7	9.2±0.6	4.6±0.3	0.36±0.01
					10	6	20	0.0	3.1	138.8±12.1	23.7±1.5	490.4±0.9	115.6±0.2	114.4±0.2	3.0±2.0	11.9±0.8	6.0±0.4	1.50±0.02
					10	6	40	0.0	3.8	185.2±19.4	32.6±2.5	440.0±0.9	111.6±0.2	110.5±0.2	3.4±2.3	7.6±0.5	3.8±0.3	1.84±0.03
					10	6	80	0.0	5.3	270.8±39.9	50.0±5.2	382.6±1.2	107.0±0.3	107.8±0.3	4.4±2.9	3.3±0.2	1.6±0.1	2.39±0.06
					10	6	10	0.5	2.8	115.8±9.0	20.9±1.1	534.4±0.9	118.6±0.2	117.8±0.2	2.9±2.0	15.3±1.1	7.6±0.5	1.29±0.02
					10	6	10	1.0	3.2	144.0±12.2	25.0±1.5	511.2±0.9	116.9±0.2	115.8±0.2	3.2±2.2	11.8±0.8	5.9±0.4	1.57±0.02
					All[23]										2.5±1.9	150.9±11.9	10.3±1.5[24]	
G	62	90	60	2438	5	6	5	0.0	2.3	72.5±9.7	14.1±1.2	549.8±1.6	119.0±0.3	118.1±0.3	2.9±2.0	16.1±1.1	8.0±0.6	0.64±0.02
					10	6	10	0.0	3.1	116.7±15.8	21.1±2.0	530.8±1.6	118.3±0.3	117.7±0.3	3.0±2.1	14.5±1.0	7.2±0.5	1.26±0.03
					15	6	15	0.0	3.9	161.2±21.0	27.0±2.5	515.5±1.6	117.9±0.4	117.4±0.4	3.1±2.1	13.8±1.0	6.9±0.5	1.88±0.04
					20	6	20	0.0	4.5	192.0±24.8	32.5±3.0	505.3±1.6	118.2±0.4	117.2±0.4	3.2±2.2	13.3±0.9	6.6±0.5	2.52±0.06
					10	3	10	0.0	4.2	156.4±18.5	27.7±2.2	553.2±1.6	121.9±0.4	121.5±0.4	3.0±2.1	18.5±1.3	9.2±0.6	2.76±0.06
					10	11	10	0.0	2.2	88.4±13.4	15.2±1.6	502.2±1.5	113.4±0.3	113.0±0.3	2.9±2.0	11.9±1.3	6.0±0.4	0.66±0.02
					10	16	10	0.0	2.1	77.6±12.6	14.1±1.5	474.5±1.5	108.9±0.4	108.3±0.3	2.8±1.9	10.0±0.7	5.0±0.3	0.47±0.01
					10	21	10	0.0	2	69.3±12.1	13.0±1.5	452.5±1.5	104.6±0.4	104.3±0.3	2.6±1.8	8.6±0.6	4.3±0.3	0.36±0.01
					10	6	20	0.0	3.4	151.1±22.5	25.7±2.7	485.3±1.5	114.5±0.4	114.3±0.4	3.1±2.1	11.1±0.8	5.5±0.4	1.50±0.04
					10	6	40	0.0	4.1	200.8±36.0	33.2±4.3	436.4±1.6	110.8±0.4	110.1±0.4	3.5±2.4	7.0±0.5	3.5±0.2	1.85±0.06
					10	6	80	0.0	6	290.3±72.3	56.1±9.8	370.9±1.9	104.2±0.5	107.2±0.6	4.4±3.0	3.2±0.2	1.6±0.1	2.41±0.11
					10	6	10	0.5	3.1	120.1±16.3	21.8±2.0	530.0±1.6	118.2±0.4	117.7±0.4	3.0±2.1	14.3±1.0	7.1±0.5	1.29±0.03
					10	6	10	1.0	3.6	150.9±22.2	27.2±2.8	508.2±1.7	116.0±0.4	115.9±0.4	3.3±2.3	11.1±0.8	5.5±0.4	1.56±0.34
					All										2.6±2.0	151.4±12.1	10.4±1.5	
G	31	90	60	2353	5	6	5	0.0	0.7	31.8±8.9	4.5±0.8	561.1±2.4	118.0±0.5	118.2±0.5	3.0±2.0	8.6±0.6	4.3±0.3	0.62±0.02
					10	6	10	0.0	1.2	66.9±17.4	9.3±1.7	542.2±2.3	118.1±0.5	118.4±0.5	3.2±2.1	7.5±0.5	3.7±0.3	1.28±0.04
					15	6	15	0.0	1.4	86.0±22.6	12.9±2.4	532.4±2.3	117.6±0.5	117.9±0.5	3.2±2.2	7.2±0.5	3.6±0.2	1.92±0.06
					20	6	20	0.0	2	108.7±28.3	16.9±3.1	517.5±2.3	117.6±0.5	117.5±0.5	3.2±2.2	6.7±0.5	3.4±0.2	2.59±0.08
					10	3	10	0.0	2.3	79.1±19.4	13.9±2.3	566.7±2.3	121.4±0.5	121.4±0.5	3.2±2.1	7.5±0.5	3.7±0.3	2.79±0.08
					10	11	10	0.0	0.7	44.8±14.0	6.5±1.3	514.5±2.3	113.4±0.5	111.9±0.5	3.0±2.0	6.0±0.4	3.0±0.2	0.67±0.02
					10	16	10	0.0	0.6	35.6±12.5	4.5±1.0	489.6±2.3	108.4±0.5	107.9±0.5	2.8±1.9	5.1±0.3	2.5±0.2	0.47±0.02
					10	21	10	0.0	0.6	29.4±11.6	4.2±1.1	465.7±2.2	104.8±0.5	103.8±0.5	2.7±1.8	4.3±0.3	2.2±0.1	0.37±0.01
					10	6	20	0.0	1.4	95.8±26.4	13.2±2.7	499.0±2.3	114.1±0.5	114.6±0.5	3.3±2.2	5.7±0.4	2.8±0.2	1.53±0.06
					10	6	40	0.0	1.6	113.4±40.6	16.1±4.4	436.0±2.3	110.5±0.6	111.7±0.6	3.7±2.5	3.6±0.2	1.8±0.1	1.87±0.09
					10	6	80	0.0	1.7	170.7±83.2	17.3±6.6	402.1±3.0	106.0±0.8	107.3±0.8	4.6±3.1	1.8±0.1	0.9±0.1	2.47±0.15
					10	6	10	0.5	1.2	68.6±17.9	9.5±1.8	542.2±2.3	118.0±0.5	118.2±0.5	3.2±2.1	7.4±0.5	3.7±0.2	1.30±0.04
					10	6	10	1.0	1.5	94.4±25.8	13.9±2.7	514.3±2.4	116.3±0.5	116.4±0.5	3.4±2.3	5.7±0.4	2.8±0.2	1.58±0.05
					All										2.7±2.3	229.3±19.5	22.6±3.5	

[1] – The shape of the point-spread function (PSF) used in the simulation

[2] – Gaussian-shaped PSF with standard deviations of 180 nm in the x & y directions and 880 nm in the z direction. This PSF is the equivalent of the Numeric PSF used in this work, when comparing the energies of both PSFs

[3] – Numerically-calculated PSF. It is based on an excitation PSF calculated using the PSFLab web server that was doubled. The convolution with the pinhole function was not considered as the simulations mimick measurements with overfilling of objective back aperture.

[4] – The calculated for 15 molecules in a rectangular box with varying volumes (see Table S1 for box dimensons).

[5] – The translational diffusion coefficients of the particles diffusing in a rectangular box.

[6] – The overall time in which 15 simulated molecules were diffusing in a rectangular box.

[7] – The background (BG) rate as was retrieved by from exponential function fitting to the slow process in the inter-photon time histogram. The simulated background from dark counts was 2300 Hz. The retrieved values were always higher than 2300 Hz, due to molecules diffusing outside the effective detection volume and emitting photons at a rate comparable to the BG rate. As the concentration decreased, so did the value of the retrieved BG rate, relative to the simulated BG rate due to dark counts.

PSF (G/N)	C (pM)	D (µm²/s)	Sim time (s)	BG rate (Hz)	m	F	sz th	w sz (ms)	% photonic impurity	Fraction of impure bursts ($x10^{-3}$)	Mean burst impurity ($x10^{-3}$)	Position dispersion – z (nm)	Position dispersion – x (nm)	Position dispersion – y (nm)	FCS diffusion time (ms)	FCS mean molecules in the PSF ($x10^{-3}$)	P(N>1) ($x10^{-3}$)	Mean burst width (ms)
G	15.5	90	60	2339	5	6	5	0.0	0.4	16.9±8.2	3.2±1.0	520.5±3.0	119.2±0.7	120.0±0.7	2.8±1.9	4.9±0.3	2.5±0.2	0.59±0.02
					10	6	10	0.0	0.6	35.0±17.5	5.4±1.9	494.7±2.9	118.9±0.7	120.9±0.7	2.9±2.0	3.9±0.3	2.0±0.1	1.27±0.06
					15	6	15	0.0	0.7	44.4±22.1	6.1±2.1	473.2±2.8	119.2±0.7	121.2±0.7	3.0±2.0	3.8±0.3	1.9±0.1	1.86±0.08
					20	6	20	0.0	0.7	47.8±25.5	7.5±2.7	464.2±2.8	118.5±0.7	120.7±0.7	3.0±2.1	3.7±0.3	1.8±0.1	2.51±0.11
					10	3	10	0.0	0.8	35.1±17.5	6.4±2.2	520.1±2.9	122.7±0.7	123.4±0.7	2.9±2.0	5.2±0.4	2.6±0.2	2.78±0.10
					10	11	10	0.0	0.4	26.1±14.6	3.8±1.4	462.0±2.8	114.5±0.7	115.4±0.7	2.8±1.9	3.2±0.2	1.6±0.1	0.67±0.03
					10	16	10	0.0	0.3	23.7±13.9	2.7±1.1	440.5±2.7	110.2±0.7	111.4±0.7	2.6±1.8	2.7±0.2	1.4±0.0	0.48±0.02
					10	21	10	0.0	0.3	21.7±13.3	2.7±1.2	417.0±2.7	106.5±0.7	105.9±0.7	2.5±1.7	2.4±0.2	1.1±0.0	0.36±0.02
					10	6	20	0.0	0.7	41.8±24.4	6.2±2.6	438.0±2.7	115.1±0.7	117.8±0.7	2.9±2.0	3.0±0.2	1.5±0.1	1.51±0.08
					10	6	40	0.0	0.8	44.8±35.5	6.5±3.4	398.5±2.9	110.1±0.8	115.0±0.8	3.3±2.3	1.9±0.1	1.0±0.0	1.82±0.12
					10	6	80	0.0	1.1	68.2±77.5	10.8±7.0	391.3±4.0	105.7±1.1	110.7±1.1	3.9±3.0	0.9±0.1	0.4±0.0	2.32±0.23
					10	6	10	0.5	0.7	36.0±17.9	5.5±2.0	492.1±2.9	118.8±0.7	120.9±0.7	2.9±2.0	3.9±0.3	1.9±0.1	1.30±0.06
					10	6	10	1.0	0.8	47.6±25.4	6.6±2.5	465.1±3.0	116.9±0.7	120.1±0.8	3.1±2.2	3.0±0.2	1.5±0.1	1.56±0.07
					All										2.5±2.3	341.8±31.7	46.7±7.7	
G	62	22.5	60	2340	5	6	5	0.0	1	48.4±10.0	9.6±1.3	594.5±1.8	119.3±0.4	120.0±0.4	9.2±6.1	15.8±1.0	7.9±0.5	0.90±0.03
					10	6	10	0.0	1.5	67.9±16.0	12.5±1.9	585.4±1.8	119.5±0.4	119.8±0.4	9.4±6.3	14.6±0.9	7.3±0.4	1.75±0.07
					15	6	15	0.0	1.6	78.5±19.6	13.5±2.3	576.7±1.7	119.8±0.4	119.8±0.4	9.8±6.5	14.4±0.9	7.2±0.4	2.50±0.10
					20	6	20	0.0	1.8	96.5±23.7	16.1±2.8	569.8±1.7	120.1±0.3	119.7±0.4	10.0±6.6	14.3±0.9	7.2±0.4	3.26±0.13
					10	3	10	0.0	1.9	79.1±19.4	15.7±2.3	609.7±1.8	123.7±0.4	124.0±0.4	10.0±6.6	17.6±1.1	8.8±0.5	3.64±0.13
					10	11	10	0.0	1	66.7±15.0	12.0±1.8	548.8±1.7	113.2±0.4	113.7±0.4	8.9±5.9	12.0±0.8	6.0±0.4	0.98±0.04
					10	16	10	0.0	0.8	59.2±13.7	10.2±1.6	519.2±1.7	108.1±0.4	108.3±0.4	8.3±5.4	10.2±0.6	5.1±0.3	0.67±0.03
					10	21	10	0.0	0.7	48.3±12.4	8.4±1.4	492.8±1.7	103.0±0.4	102.7±0.4	7.3±4.8	8.7±0.5	4.3±0.3	0.50±0.02
					10	6	20	0.0	1.5	73.3±20.7	13.0±2.4	551.9±1.7	115.6±0.4	116.4±0.4	9.5±6.3	12.5±0.8	6.2±0.4	2.18±0.09
					10	6	40	0.0	1.6	85.5±28.0	14.2±3.2	510.4±1.7	111.9±0.4	112.3±0.4	9.8±6.5	10.0±0.6	5.0±0.3	2.63±0.12
					10	6	80	0.0	1.8	105.3±40.1	18.2±4.8	469.7±1.7	109.1±0.4	107.9±0.4	10.4±6.9	7.4±0.5	3.7±0.2	3.17±0.16
					10	6	10	0.5	1.5	68.3±16.1	12.5±1.9	585.6±1.8	119.4±0.4	119.7±0.4	9.5±6.3	14.6±0.9	7.3±0.4	1.75±0.07
					10	6	10	1.0	1.5	71.5±18.6	13.3±2.3	572.0±1.8	117.4±0.4	117.8±0.4	9.6±6.4	13.3±0.8	6.6±0.4	2.02±0.08
					All										8.9±6.8	167.2±11.9	12.5±1.7	
G	62	5.625	180	2334	5	6	5	0.0	0.9	34.9±6.1	6.2±0.7	582.6±1.0	117.2±0.2	119.2±0.2	35.1±22.7	13.8±0.8	6.9±0.4	1.21±0.06
					10	6	10	0.0	1	58.1±12.1	7.6±1.2	574.4±1.0	116.8±0.2	118.5±0.2	36.0±23.3	12.7±0.7	6.3±0.3	2.76±0.14
					15	6	15	0.0	1.1	67.2±15.2	7.4±1.4	568.9±1.0	116.8±0.2	118.8±0.2	36.1±23.3	12.5±0.7	6.2±0.3	3.95±0.20
					20	6	20	0.0	1.1	75.8±17.9	8.0±1.6	565.6±1.0	117.0±0.2	118.7±0.2	36.5±23.5	12.4±0.7	6.2±0.3	5.01±0.25
					10	3	10	0.0	1.3	61.9±13.7	7.3±1.3	599.2±1.1	121.2±0.2	123.5±0.2	37.9±24.6	14.7±0.8	7.3±0.4	5.29±0.25
					10	11	10	0.0	0.9	49.6±9.5	8.0±1.0	542.5±1.0	110.7±0.2	112.5±0.2	32.7±21.0	10.8±0.6	5.4±0.3	1.35±0.07
					10	16	10	0.0	0.8	43.4±8.2	7.2±0.9	516.2±1.0	105.2±0.2	106.5±0.2	30.1±19.2	9.3±0.5	4.6±0.3	0.86±0.04
					10	21	10	0.0	0.7	42.2±7.7	6.7±0.8	491.8±1.0	99.6±0.2	101.5±0.2	26.9±17.2	7.9±0.4	3.9±0.2	0.61±0.03
					10	6	20	0.0	1.1	80.0±17.6	9.3±1.6	554.9±1.0	114.2±0.2	115.6±0.2	34.8±22.5	11.5±0.6	5.7±0.3	3.78±0.19
					10	6	40	0.0	1.1	99.7±23.4	11.8±2.3	535.8±1.0	111.0±0.2	113.2±0.2	34.1±22.0	10.4±0.6	5.2±0.3	4.74±0.24
					10	6	80	0.0	1.1	110.8±30.3	11.1±2.6	511.3±1.0	107.3±0.2	109.6±0.2	33.6±21.7	8.9±0.5	4.4±0.2	5.92±0.31
					10	6	10	0.5	1	57.7±12.1	7.3±1.2	574.2±1.0	116.7±0.2	118.5±0.2	35.9±23.2	12.7±0.7	6.3±0.3	2.77±0.14
					10	6	10	1.0	1	66.1±14.2	7.7±1.3	566.2±1.0	115.4±0.2	117.4±0.2	35.4±22.8	12.2±0.7	6.1±0.3	3.20±0.16
					All										35.5±26.4	166.1±10.6	12.6±1.5	

[8] – The burst search time window of *m* consecutive photons

[9] – A number setting the threshold on the minimal multiplier of the photon rate, relative to the BG rate, above which, photons are considered part of a burst

[10] – The minimal amount of photons in a burst (its minimal size)

[11] – The minimal duration (width) of a burst from its first photon timestamp, till its last.

[12] – All the photons in all bursts that originated from molecules other than the main ones in bursts. No error values were calculated as it was simply a ratio of photons relative to all photons.

[13] – The fraction of photon bursts that included photons originating from molecules other than the main ones in bursts. Error values are based on the calculation of a 95% confidence interval.

[14] – The mean of the fraction of photons originating from molecules other than the main ones per burst, for all bursts. Error values are based on the calculation of the standard error.

[15] – The standard deviation of all molecule positions when emitting a photon, in the z coordinate. Error values are based on the calculation of the uncertainty of the standard error values.

[16] - The standard deviation of all molecule positions when emitting a photon, in the x coordinate. Error values are based on the calculation of the uncertainty of the standard error values.

[17] - The standard deviation of all molecule positions when emitting a photon, in the y coordinate. Error values are based on the calculation of the uncertainty of the standard error values.

[18] – The best fit value of the diffusion time through the effective detection volume, as it was recovered from a fit of a fluorescence autocorrelation model of 3D diffusion, to the photon timestamp autocorrelation functions. Error values are the fitting errors.

PSF (G/N)	C (pM)	D (μm^2/s)	Sim time (s)	Bg rate (Hz)	m	F	Sz th	W sz (ms)	% photonic impurity	Fraction of impure bursts (x10^{-3})	Mean burst impurity (x10^{-3})	Position dispersion – z (nm)	Position dispersion – x (nm)	Position dispersion – y (nm)	FCS diffusion time (ms)	FCS mean molecules in the PSF (x10^{-3})	P(N>1) (x10^{-3})	Mean burst width (ms)
G	62	5.625	60	2338	5	6	5	0.0	0.7	41.8±11.4	7.5±1.4	602.3±1.8	117.5±0.4	118.9±0.4	33.0±21.5	15.4±0.9	7.7±0.4	1.26±0.11
					10	6	10	0.0	0.8	65.4±21.3	7.5±1.9	595.1±1.8	117.5±0.4	117.9±0.4	33.6±22.0	14.2±0.8	7.1±0.4	2.81±0.23
					15	6	15	0.0	0.9	85.3±28.4	9.7±2.5	590.9±1.8	117.4±0.4	118.3±0.4	33.8±22.1	14.1±0.8	7.0±0.4	4.00±0.32
					20	6	20	0.0	0.9	78.9±29.8	8.2±2.7	592.0±1.8	117.2±0.4	118.7±0.4	34.5±22.5	14.2±0.8	7.1±0.4	4.96±0.38
					10	3	10	0.0	0.9	71.3±24.7	7.9±2.0	615.8±1.8	121.3±0.4	123.5±0.4	35.5±23.3	16.4±0.9	8.2±0.5	5.36±0.40
					10	11	10	0.0	0.7	42.6±15.0	5.7±1.5	566.2±1.8	110.8±0.3	112.2±0.3	30.2±19.7	12.1±0.7	6.0±0.3	1.42±0.12
					10	16	10	0.0	0.7	36.6±12.5	6.5±1.5	536.9±1.7	106.1±0.3	107.4±0.3	27.9±18.2	10.4±0.6	5.2±0.3	0.87±0.07
					10	21	10	0.0	0.6	35.6±11.6	4.9±1.1	514.5±1.7	101.2±0.3	102.3±0.3	24.8±16.1	8.9±0.5	4.5±0.3	0.61±0.05
					10	6	20	0.0	0.8	79.2±28.8	8.2±2.5	581.6±1.8	114.1±0.3	115.6±0.4	32.3±21.1	13.0±0.7	6.5±0.4	3.75±0.31
					10	6	40	0.0	0.8	97.8±39.1	9.7±3.4	563.3±1.8	111.1±0.3	112.3±0.4	31.8±20.7	11.6±0.7	5.8±0.3	4.85±0.40
					10	6	80	0.0	0.7	104.9±47.7	7.5±3.5	537.0±1.7	109.0±0.4	110.3±0.4	31.4±20.4	10.3±0.6	5.1±0.3	5.76±0.49
					10	6	10	0.5	0.8	65.4±21.3	7.5±1.9	595.1±1.8	117.5±0.4	117.9±0.4	33.6±22.0	14.2±0.8	7.1±0.4	2.81±0.23
					10	6	10	1.0	0.8	78.0±25.7	8.8±2.2	589.9±1.8	116.0±0.4	116.6±0.4	32.9±21.6	13.7±0.8	6.8±0.4	3.26±0.27
					All										33.0±24.7	16.6±10.6	12.3±1.5	
N	62	90	180	2483	5	6	5	0.0	2.1	133.4±9.2	25.6±1.2	466.3±1.3	134.6±0.4	134.0±0.4	1.7±1.1	7.8±0.6	3.9±0.3	0.50±0.01
					10	6	10	0.0	2.7	228.3±16.7	27.7±1.6	411.1±1.2	121.6±0.3	118.4±0.3	1.8±1.2	5.9±0.4	2.9±0.2	1.07±0.02
					15	6	15	0.0	3.2	304.4±21.7	32.4±1.9	407.5±1.2	126.9±0.4	122.9±0.4	1.8±1.2	5.4±0.4	2.7±0.2	1.65±0.03
					20	6	20	0.0	3.6	352.1±25.7	37.0±2.4	402.8±1.3	129.0±0.4	124.7±0.4	1.9±1.3	4.9±0.3	2.5±0.2	2.20±0.03
					10	3	10	0.0	3.9	356.5±18.0	50.4±2.0	500.3±1.3	156.2±0.4	154.6±0.4	1.8±1.2	9.6±0.7	4.8±0.4	2.45±0.03
					10	11	10	0.0	1.9	149.9±14.3	18.4±1.3	362.7±1.1	102.0±0.3	99.1±0.3	1.7±1.1	4.3±0.3	2.2±0.2	0.54±0.01
					10	16	10	0.0	1.7	120.7±13.4	14.2±1.2	338.8±1.1	94.5±0.3	92.4±0.3	1.6±1.1	3.4±0.2	1.7±0.1	0.37±0.01
					10	21	10	0.0	1.5	100.6±12.7	11.9±1.1	321.9±1.1	90.2±0.3	85.5±0.3	1.6±1.0	2.9±0.2	1.4±0.1	0.28±0.01
					10	6	20	0.0	2.9	265.1±24.7	27.3±2.2	363.9±1.2	112.7±0.4	108.8±0.4	1.9±1.3	3.7±0.3	1.8±0.1	1.30±0.03
					10	6	40	0.0	3.8	315.6±45.1	36.3±4.6	316.8±1.4	105.2±0.5	96.7±0.4	2.3±1.6	1.7±0.1	0.8±0.1	1.65±0.04
					10	6	80	0.0	7	444.4±136.9	72.8±18.8	286.0±2.7	93.3±0.9	85.9±0.8	3.1±2.3	0.3±0.0	0.2±0.0	2.20±0.15
					10	6	10	0.5	2.7	234.1±17.2	28.5±1.6	411.6±1.2	121.9±0.4	118.8±0.3	1.8±1.2	5.7±0.4	2.9±0.2	1.10±0.02
					10	6	10	1.0	3.2	270.5±24.5	31.9±2.3	386.6±1.3	116.5±0.4	111.6±0.4	2.0±1.3	3.8±0.3	1.9±0.1	1.38±0.02
					All										1.5±1.3	327.6±31.0	43.3±7.3	
N	62	90	60	2474	5	6	5	0.0	2	129.0±16.2	23.5±2.0	458.9±2.2	134.3±0.7	133.7±0.7	1.6±1.1	7.1±0.5	3.6±0.3	0.50±0.01
					10	6	10	0.0	2.6	232.2±30.7	25.2±2.5	402.4±2.1	122.7±0.6	114.5±0.6	1.6±1.2	5.3±0.4	2.6±0.2	1.10±0.03
					15	6	15	0.0	3	303.4±39.1	29.5±3.2	400.0±2.1	125.6±0.7	121.6±0.6	1.7±1.2	4.9±0.4	2.4±0.2	1.65±0.05
					20	6	20	0.0	3.4	358.4±47.3	34.4±4.1	391.9±2.2	124.5±0.7	124.3±0.7	1.8±1.2	4.3±0.3	2.1±0.2	2.23±0.07
					10	3	10	0.0	3.6	361.7±32.0	49.5±3.4	501.6±2.4	160.8±0.8	160.2±0.8	1.7±1.2	9.0±0.7	4.5±0.3	2.45±0.05
					10	11	10	0.0	2.1	154.4±26.2	18.0±2.2	357.9±2.0	105.5±0.6	98.3±0.5	1.6±1.1	3.9±0.3	1.9±0.1	0.55±0.02
					10	16	10	0.0	2	124.1±24.6	13.9±2.0	329.7±1.9	95.1±0.6	92.1±0.5	1.5±1.1	3.1±0.2	1.5±0.1	0.37±0.01
					10	21	10	0.0	1.9	105.7±23.5	13.3±2.1	309.2±1.9	86.7±0.5	84.6±0.5	1.5±1.0	2.5±0.2	1.3±0.1	0.28±0.01
					10	6	20	0.0	2.8	261.0±45.3	22.8±3.4	355.9±2.1	112.7±0.7	105.5±0.6	1.8±1.2	3.3±0.2	1.6±0.1	1.35±0.05
					10	6	40	0.0	4	357.7±85.9	33.4±8.0	317.4±2.5	104.0±0.8	99.2±0.8	2.3±1.6	1.5±0.1	0.8±0.1	1.76±0.09
					10	6	80	0.0	7.8	521.7±220.9	73.1±29.6	329.2±4.8	108.5±1.6	115.4±1.6	4.0±2.7	0.5±0.0	0.2±0.0	2.21±0.25
					10	6	10	0.5	2.7	240.7±31.8	26.2±2.6	401.9±2.1	123.5±0.6	115.2±0.6	1.7±1.2	5.1±0.4	2.6±0.2	1.14±0.03
					10	6	10	1.0	3.1	291.0±44.6	30.2±3.8	386.2±2.3	123.1±0.7	112.9±0.7	1.8±1.3	3.5±0.3	1.8±0.1	1.41±0.04
					All										1.4±1.3	327.9±32.4	43.3±7.7	

[19] - The best fit value of the average number of molecules traversing the effective detection volume at any given moment, as it was recovered from a fit of a fluorescence autocorrelation model of 3D diffusion, to the photon timestamp autocorrelation functions. Error values are the fitting errors.

[20] – The probability of having more than 1 molecule traverse the beam. It was calculated out of the best fit value of the average number of molecules traversing the effective detection volume at any moments. The calculation was performed differently for all photons and for photons in bursts (see explanations 22 & 24). The error values are propagated from the error values of the average number of molecules in the effective detection volume.

[21] – The mean of all burst widths.

[22] – The photon timestamp autocorrelation function was based only on burst photons, hence on events of at least 1 molecule. Therefore, the probability of having >1 molecule was calculated as the Poisson probability of >1 molecule, relative to the Poisson probability of having >0 molecule

[23] – The FCS calculations were performed for all photons, including ones that were outside bursts.

[24] - The photon timestamp autocorrelation function was based only all photons, hence on events of 0 molecules (background) or more. Therefore, the probability of having >1 molecule was calculated as the Poisson probability of >1 molecule

PSF (G/N)	C (pM)	D (μm²/s)	Sim time (s)	Bg rate (Hz)	m	F	Sz th	W sz (ms)	% photonic impurity	Fraction of impure bursts (x10^{-3})	Mean burst impurity (x10^{-3})	Position dispersion – z (nm)	Position dispersion – x (nm)	Position dispersion – y (nm)	FCS diffusion time (ms)	FCS mean molecules in the PSF (x10^{-3})	P(N>1) (x10^{-3})	Mean burst width (ms)
N	31	90	60	2382	5	6	5	0.0	0.8	63.9±15.4	11.7±1.8	422.2±2.8	120.7±0.8	121.2±0.8	1.5±1.0	4.1±0.3	2.1±0.2	0.49±0.02
					10	6	10	0.0	0.9	116.2±31.0	11.8±2.3	384.8±2.7	105.4±0.7	110.7±0.8	1.6±1.1	3.0±0.2	1.5±0.1	1.10±0.04
					15	6	15	0.0	1.2	152.5±41.2	14.0±2.9	363.3±2.6	104.0±0.8	104.6±0.8	1.6±1.1	2.7±0.2	1.4±0.1	1.69±0.07
					20	6	20	0.0	1.3	169.5±48.2	15.6±3.5	363.3±2.7	105.6±0.8	107.0±0.8	1.7±1.2	2.5±0.2	1.3±0.1	2.25±0.09
					10	3	10	0.0	1.5	163.5±33.3	21.0±3.1	455.1±3.0	133.9±0.9	136.3±0.9	1.7±1.1	5.0±0.4	2.5±0.2	2.52±0.07
					10	11	10	0.0	0.7	70.4±25.2	75.4±1.9	337.8±2.5	89.4±0.7	92.3±0.7	1.6±1.1	2.2±0.2	1.1±0.1	0.56±0.02
					10	16	10	0.0	0.6	46.2±20.9	50.3±1.5	314.6±2.5	84.7±0.7	82.6±0.7	1.6±1.0	1.8±0.1	0.9±0.1	0.38±0.02
					10	21	10	0.0	0.5	43.8±20.5	6.8±2.0	303.2±2.5	82.2±0.7	79.8±0.7	1.5±1.0	1.5±0.1	0.8±0.1	0.29±0.01
					10	6	20	0.0	1	137.6±46.1	11.3±2.8	340.4±2.7	95.8±0.8	98.7±0.8	1.7±1.2	1.9±0.1	1.0±0.1	1.31±0.06
					10	6	40	0.0	1	147.1±86.3	10.5±5.6	289.7±3.3	86.0±1.0	84.0±0.9	2.3±1.6	0.9±0.1	0.5±0.0	1.67±0.13
					10	6	80	0.0	0.9	200.0±301.6	8.1±7.0	249.9±5.8	85.1±2.0	86.2±2.0	3.3±2.1	0.2±0.0	0.1±0.0	2.20±0.54
					10	6	10	0.5	0.9	121.2±32.3	12.3±2.4	385.8±2.7	105.6±0.8	111.1±0.8	1.6±1.1	2.9±0.2	1.5±0.1	1.13±0.04
					10	6	10	1.0	1.1	157.9±47.7	15.6±3.5	371.3±3.1	102.3±0.8	106.7±0.9	1.7±1.2	2.0±0.1	1.0±0.1	1.39±0.05
					All										1.3±1.4	518.7±58.4	95.9±18.0	
N	15.5	90	60	2358	5	6	5	0.0	0.5	24.4±12.2	6.0±1.8	481.4±4.8	142.3±1.4	127.4±1.3	1.7±1.1	2.6±0.2	1.3±1.2	0.46±0.02
					10	6	10	0.0	0.8	52.6±32.0	8.6±3.7	417.4±4.4	115.0±1.2	107.2±1.1	1.7±1.1	1.3±0.1	0.7±0.6	1.08±0.06
					15	6	15	0.0	1	75.8±45.7	11.4±5.2	377.4±4.2	117.5±1.3	108.5±1.2	1.8±1.1	1.2±0.1	0.6±0.6	1.73±0.09
					20	6	20	0.0	1	90.0±57.1	11.2±5.9	349.1±4.0	108.0±1.2	106.3±1.2	1.9±1.2	1.1±0.1	0.6±0.5	2.36±0.14
					10	3	10	0.0	0.8	78.3±35.0	9.8±3.2	475.8±4.7	134.8±1.3	134.4±1.3	1.8±1.1	2.4±0.2	1.2±1.1	2.50±0.10
					10	11	10	0.0	0.6	33.3±26.5	6.9±3.5	327.0±3.7	86.4±1.0	90.8±1.0	1.5±1.0	1.0±0.1	0.5±0.4	0.55±0.03
					10	16	10	0.0	0.4	18.8±21.2	3.1±2.7	296.0±3.6	79.8±1.0	82.3±1.0	1.4±0.9	0.7±0.0	0.4±0.3	0.39±0.03
					10	21	10	0.0	0.3	19.7±22.4	3.4±2.7	280.4±3.5	77.0±1.0	78.1±1.0	1.4±0.9	0.6±0.0	0.3±0.3	0.30±0.02
					10	6	20	0.0	0.9	64.5±50.9	10.4±6.1	319.1±3.9	98.2±1.2	95.8±1.2	1.9±1.2	0.8±0.1	0.4±0.4	1.29±0.07
					10	6	40	0.0	0.3	71.4±101.7	3.6±3.1	262.0±4.8	91.0±1.7	84.0±1.5	2.4±1.6	0.3±0.0	0.2±0.2	1.58±0.17
					10	6	80	0.0	0.3			195.6±8.8	74.2±3.4	80.9±3.7				1.59±1.42
					10	6	10	0.5	0.8	55.6±33.8	9.1±4.0	423.8±4.6	116.3±1.2	107.9±1.2	1.7±1.1	1.3±0.1	0.6±0.6	1.12±0.06
					10	6	10	1.0	0.9	66.0±48.1	10.0±5.4	377.3±4.7	102.0±1.3	105.1±1.3	1.8±1.1	0.9±0.1	0.4±0.4	1.36±0.06
					All										1.5±2.0	1059±150.9	285.9±55.2	
N	62	22.5	60	2456	5	6	5	0.0	1.2	125.3±19.1	24.1±2.4	472.2±2.3	125.9±0.6	125.8±0.6	5.2±3.2	7.1±0.4	3.5±3.3	0.64±0.03
					10	6	10	0.0	1.3	190.2±35.7	21.4±3.2	415.9±2.1	106.1±0.5	105.0±0.5	5.4±3.4	5.6±0.3	2.8±2.6	1.45±0.07
					15	6	15	0.0	1.3	223.5±43.4	18.2±3.2	397.9±2.0	104.7±0.5	104.8±0.5	5.6±3.5	5.4±0.3	2.7±2.5	2.07±0.09
					20	6	20	0.0	1.7	274.0±51.5	21.0±3.8	395.3±2.0	109.4±0.6	108.2±0.5	5.7±3.6	5.3±0.3	2.6±2.5	2.73±0.12
					10	3	10	0.0	2.1	307.1±39.7	34.6±3.7	496.1±2.3	139.9±0.6	136.3±0.6	5.8±3.7	8.4±0.5	4.2±3.9	3.04±0.12
					10	11	10	0.0	0.7	125.5±30.3	11.8±2.2	360.9±1.9	89.4±0.5	91.3±0.5	5.0±3.1	4.3±0.3	2.1±2.0	0.82±0.04
					10	16	10	0.0	0.6	94.9±25.9	9.6±2.0	344.7±1.9	86.5±0.5	86.6±0.5	4.7±3.0	3.6±0.2	1.8±1.7	0.56±0.03
					10	21	10	0.0	0.4	74.1±23.3	7.1±1.7	323.0±1.9	78.7±0.5	80.3±0.5	4.2±2.6	3.0±0.2	1.5±1.4	0.43±0.02
					10	6	20	0.0	1.2	196.6±45.6	15.8±3.4	372.9±2.0	98.4±0.5	96.2±0.5	5.3±3.4	4.5±0.3	2.2±2.1	1.76±0.08
					10	6	40	0.0	1.3	222.9±62.3	18.1±5.2	346.2±2.0	93.8±0.5	91.0±0.5	5.6±3.6	3.3±0.2	1.7±1.6	2.10±0.11
					10	6	80	0.0	0.9	208.3±96.1	11.7±7.2	292.7±2.1	82.6±0.6	83.6±0.6	6.6±4.0	1.8±0.1	0.9±0.9	2.60±0.19
					10	6	10	0.5	1.3	191.4±35.9	21.5±3.2	415.9±2.1	106.1±0.5	105.0±0.5	5.4±3.4	5.6±0.3	2.8±2.6	1.46±0.07
					10	6	10	1.0	1.3	195.1±43.1	18.0±3.5	393.5±2.1	100.9±0.5	99.2±0.5	5.6±3.4	4.7±0.3	2.3±2.2	1.72±0.08
					All										5.0±4.2	337.8±27.5	45.7±6.6	

PSF (G/N)	C (pM)	D ($\mu m^2/s$)	Sim time (s)	Bg rate (Hz)	m	F	Sz th	W sz (ms)	% photonic impurity	Fraction of impure bursts ($x10^{-3}$)	Mean burst impurity ($x10^{-3}$)	Position dispersion – z (nm)	Position dispersion – x (nm)	Position dispersion – y (nm)	FCS diffusion time (ms)	FCS mean molecules in the PSF ($x10^{-3}$)	P(N>1) ($x10^{-3}$)	Mean burst width (ms)
N	62	5.625	180	2431	5	6	5	0.0	0.7	105.2±11.6	21.4±1.6	460.2±1.3	127.0±0.4	126.2±0.4	19.1±12.2	6.4±0.4	3.2±0.2	0.75±0.04
					10	6	10	0.0	0.7	189.8±27.1	15.4±2.1	407.5±1.2	103.8±0.3	102.3±0.3	19.8±12.6	4.9±0.3	2.4±0.1	2.02±0.12
					15	6	15	0.0	0.7	247.3±36.0	11.2±1.8	393.1±1.2	102.7±0.3	102.1±0.3	205±13.2	4.7±0.3	2.4±0.1	2.99±0.17
					20	6	20	0.0	0.8	291.7±43.0	12.2±2.2	385.9±1.1	102.8±0.3	101.9±0.3	20.7±13.2	4.7±0.3	2.3±0.1	3.94±0.24
					10	3	10	0.0	1.1	295.2±27.5	37.5±2.9	497.3±1.4	137.0±0.4	136.4±0.4	21.6±13.8	7.4±0.4	3.7±0.2	3.65±0.15
					10	11	10	0.0	0.5	134.4±23.0	8.1±1.2	349.4±1.1	91.1±0.3	92.3±0.3	18.2±11.6	3.9±0.2	1.9±0.1	1.16±0.07
					10	16	10	0.0	0.4	103.9±19.8	6.7±1.1	325.6±1.0	85.0±0.3	86.5±0.3	16.2±10.3	3.3±0.2	1.6±0.1	0.79±0.05
					10	21	10	0.0	0.4	83.3±17.2	5.2±1.0	308.0±1.0	79.7±0.3	81.9±0.3	15.0±9.5	2.9±0.2	1.4±0.1	0.58±0.04
					10	6	20	0.0	0.6	251.7±41.0	11.5±2.3	363.4±1.1	97.0±0.3	96.1±0.3	19.2±12.3	4.1±0.2	2.1±0.1	2.94±0.18
					10	6	40	0.0	0.6	274.8±50.6	8.9±2.2	339.2±1.1	92.7±0.3	92.7±0.3	18.9±12.0	3.6±0.2	1.8±0.1	3.56±0.22
					10	6	80	0.0	0.5	304.8±66.6	6.0±1.7	314.2±1.0	87.8±0.3	87.1±0.3	19.6±12.4	3.0±0.2	1.5±0.1	4.39±0.29
					10	6	10	0.5	0.7	190.1±27.2	15.4±2.1	407.6±1.2	103.7±0.3	102.3±0.3	19.8±12.6	4.9±0.3	2.4±0.1	2.02±0.12
					10	6	10	1.0	0.7	220.5±33.7	13.8±2.3	387.6±1.1	100.1±0.3	98.5±0.3	19.4±12.3	4.5±0.3	2.2±0.1	2.48±0.14
					All										20.6±17.4	327.3±24.5	43.2±5.7	
N	62	5.625	60	2429	5	6	5	0.0	0.7	108.6±20.3	22.7±2.7	468.3±2.2	123.0±0.6	129.5±0.6	18.9±12.0	7.1±0.4	3.6±0.2	0.77±0.06
					10	6	10	0.0	0.7	162.5±43.2	16.4±3.6	405.2±2.0	103.7±0.5	104.0±0.5	19.6±12.4	5.6±0.3	2.8±0.2	2.08±0.21
					15	6	15	0.0	0.7	195.7±57.9	15.3±4.1	383.5±1.9	98.7±0.5	101.3±0.5	20.1±12.7	5.3±0.3	2.6±0.2	3.18±0.33
					20	6	20	0.0	0.7	211.9±65.9	11.6±3.7	379.9±1.9	99.8±0.5	101.9±0.5	20.4±12.9	5.3±0.3	2.6±0.1	4.02±0.41
					10	3	10	0.0	1.1	267.5±44.4	31.9±4.0	513.4±2.3	134.8±0.6	138.3±0.6	21.6±13.7	8.5±0.5	4.2±0.2	3.66±0.25
					10	11	10	0.0	0.4	101.9±33.6	7.0±1.8	346.9±1.8	87.3±0.4	91.7±0.5	17.5±11.2	4.4±0.3	2.2±0.1	1.14±0.12
					10	16	10	0.0	0.3	60.5±25.2	3.6±1.1	317.1±1.7	81.5±0.4	86.0±0.5	16.0±10.2	3.7±0.2	1.9±0.1	0.76±0.08
					10	21	10	0.0	0.2	50.4±22.8	3.3±1.0	294.7±1.7	78.1±0.4	83.9±0.5	14.1±9.0	3.1±0.2	1.6±0.1	0.57±0.06
					10	6	20	0.0	0.6	166.7±59.1	8.9±3.3	359.1±1.8	93.8±0.5	97.8±0.5	19.2±12.2	4.7±0.3	2.4±0.1	3.01±0.32
					10	6	40	0.0	0.6	211.5±79.8	8.3±2.8	336.1±1.8	88.4±0.5	96.3±0.5	18.8±11.9	4.1±0.2	2.1±0.1	3.77±0.41
					10	6	80	0.0	0.5	205.9±98.6	8.0±3.9	299.1±1.7	82.4±0.5	91.2±0.5	19.3±12.4	3.4±0.2	1.7±0.1	4.58±0.52
					10	6	10	0.5	0.7	162.5±43.2	16.4±3.6	405.2±2.0	103.4±0.5	104.0±0.5	19.4±12.2	5.6±0.3	2.8±0.2	2.08±0.21
					10	6	10	1.0	0.6	172.6±53.2	11.9±3.1	377.9±1.9	96.2±0.5	99.5±0.5	19.3±12.2	5.0±0.3	2.5±0.1	2.63±0.27
					All										19.8±16.8	340.9±25.6	46.4±6.2	

Table S3: the values of all quantities assessed in this work for all simulations of single-molecule FRET with a mixture of two FRET subpopulations

PSF[1] (G[2]/N[3])	Sim time[4] (s)	Donor ch. BG rate[5] (Hz)	Acceptor ch. BG rate[6] (Hz)	m[7]	F[8]	sz th[9]	All parameters free to change[10]						f=0.6666 fixed[11]				
							μ_1[12] (x10^3)	σ_1[13] (x10^3)	μ_2[14] (x10^3)	σ_2[15] (x10^3)	f_1[16] (x10^3)	Amplitude[17]	μ_1 (x10^3)	σ_1 (x10^3)	μ_2 (x10^3)	σ_2 (x10^3)	Amplitude
G	60	1551	829	10	6	10	729.0±7.5	84.9±6.0	492.8±13.9	92.8±11.5	634.3±16.2	167±6	722.0±6.7	90.9±6.0	480.8±11.3	82.2±9.7	1587±39
				10	11	10	727.8±6.7	92.5±6.8	476.9±9.6	74.4±9.0	617.2±18.4	183±8	727.8±6.7	92.5±6.8	476.9±9.6	74.4±9.0	1701±53
				10	21	10	741.8±9.4	87.6±7.4	491.4±18.1	101.5±15.2	632.9±19.5	170±7	733.0±8.5	96.0±7.7	476.2±14.6	87.3±12.6	1708±51
G	180	1551	852	10	6	10	730.9±3.9	83.1±3.2	486.9±8.5	98.1±7.4	658.3±8.9	507±10	725.5±3.7	88.0±3.3	476.2±7.2	87.5±6.5	4856±73
				10	11	10	732.8±5.8	87.2±5.1	478.4±11.8	92.2±10.7	664.3±13.8	536±17	731.0±5.5	88.9±5.1	475.0±11.0	88.5±10.1	5173±123
				10	21	10	739.9±10.4	92.2±7.9	487.3±23.2	103.5±18.4	669.2±22.6	495±22	736.8±9.7	94.8±7.9	480.6±20.7	98.0±16.9	5150±152
N	60	1585	902	10	6	10	719.5±11.9	93.2±9.4	465.9±22.6	99.0±18.3	644.6±24.3	74±4	714.5±10.8	97.4±9.3	457.3±19.3	91.0±16.1	769.1±26.5
				10	11	10	758.7±18.1	81.2±14.5	526.3±49.7	134.0±36.2	568.2±77.7	66±8	728.8±19.8	105.0±16.4	465.6±32.0	92.9±24.6	737.8±36.1
				10	21	10	742.1±11.2	86.1±9.6	476.7±23.5	104.2±21.6	649.4±25.0	65±4	734.6±10.1	93.9±9.8	462.6±18.7	89.1±17.7	647±29
N	180	1585	902	10	6	10	736.1±13.6	91.1±8.5	500.8±33.9	118.9±23.0	637.5±43.3	212±13	724.0±11.7	98.8±8.7	475.1±23.5	98.6±17.2	2321±63
				10	11	10	730.5±10.0	96.8±8.2	480.5±18.8	92.2±14.9	660.0±21.6	218±9	730.9±10.2	96.5±8.2	481.3±19.1	93.0±15.1	2258±64
				10	21	10	735.9±9.3	96.1±8.2	471.7±19.0	94.4±16.3	672.7±20.6	201±9	737.1±9.6	95.1±8.2	473.9±19.9	96.7±17.0	2088±68

[1] – The shape of the point-spread function (PSF) used in the simulation

[2] – Gaussian-shaped PSF with standard deviations of 180 nm in the x & y directions and 880 nm in the z direction. This PSF is the equivalent of the Numeric PSF used in this work, when comparing the energies of both PSFs

[3] – Numerically-calculated PSF. It is based on an excitation PSF calculated using the PSFLab web server that was doubled. The convolution with the pinhole function was not considered as the simulations mimick measurements with overfilling of objective back aperture.

[4] – The overall time in which 15 simulated molecules were diffusing in a rectangular box.

[5] – The background (BG) rate in the donor detection channel, as was retrieved by from exponential function fitting to the slow process in the inter-photon time histogram. The simulated background from dark counts was 1500 Hz. The retrieved values were always higher than 2300 Hz, due to molecules diffusing outside the effective detection volume and emitting photons at a rate comparable to the BG rate. As the concentration decreased, so did the value of the retrieved BG rate, relative to the simulated BG rate due to dark counts.

[6] - The background (BG) rate in the acceptor detection channel, as was retrieved by from exponential function fitting to the slow process in the inter-photon time histogram. The simulated background from dark counts was 800 Hz. The retrieved values were always higher than 2300 Hz, due to molecules diffusing outside the effective detection volume and emitting photons at a rate comparable to the BG rate. As the concentration decreased, so did the value of the retrieved BG rate, relative to the simulated BG rate due to dark counts.

[7] – The burst search time window of *m* consecutive photons

[8] – A number setting the threshold on the minimal multiplier of the photon rate, relative to the BG rate, above which, photons are considered part of a burst

[9] – The minimal amount of photons in a burst (its minimal size)

[10] – fitting to a sum-of-two-gaussians model, with all free parameters unconstrained

[11] - fitting to a sum-of-two-gaussians model, with all free parameters unconstrained, except for the fraction of the population with FRET efficiency of 0.75, that was fixed at the simulated value, f=0.6666

[12] – The best fit value of the mean FRET efficiency of the 1st population out of two (the simulated mean FRET efficiency was 0.75). The reported error values are the fitting error values.

[13] - The best fit value of the FRET efficiency standard deviation of the 1st population out of two. The reported error values are the fitting error values.

[14] – The best fit value of the mean FRET efficiency of the 2nd population out of two (the simulated mean FRET efficiency was 0.50). The reported error values are the fitting error values.

[15] - The best fit value of the FRET efficiency standard deviation of the 2nd population out of two. The reported error values are the fitting error values.

[16] – The best fit value of the fraction of the 1st population out of two (the simulated fraction was 0.666). The reported error values are the fitting error values.

[17] – The best fit value of the amplitude, as the amount of bursts. The reported error values are the fitting error values.

Table S4: the values of all quantities assessed in this work from burst analyses of single-molecule FRET measurements of a mixture of two FRET subpopulations

Sample name	m[1]	f[2]	sz th[3]	Donor ch. BG rate[4] (Hz)	Acceptor ch. BG rate[5] (Hz)	μ_1[6] (x10^3)	σ_1[7] (x10^3)	μ_2[8] (x10^3)	σ_2[9] (x10^3)
(-8)TA-(+4)NTD[10]	10	6	10	176.6±1.4	121.7±1.0	679.9±8.3	128.1±8.3	-[11]	-
	10	11	10			683.3±7.7	133.8±7.7	-	-
	10	16	10			683.7±5.0	113.1±5.0	-	-
	10	21	10			686.1±4.2	117.3±4.2	-	-
(-15)TA-(+2)NTD	10	6	10	254.7±2.1	151.1±1.1	-	-	332.9±2.1	94.7±2.1
	10	11	10			-	-	336.6±2.1	96.6±2.1
	10	16	10			-	-	336.1±3.0	90.1±3.0
	10	21	10			-	-	335.1±3.1	92.9±3.1
Mixture[12]	10	6	10	339.0±6.4	201.3±3.8	640.3±23.3	135.6±20.6	327.6±4.2	100.8±3.4
	10	11	10			648.8±25.8	142.0±23.2	330.5±4.7	99.4±3.8
	10	16	10			667.9±19.9	117.2±19.6	337.4±4.5	104.3±4.2
	10	21	10			684.4±18.9	102.7±19.8	340.7±4.9	104.0±5.1

[1] – The burst search time window of *m* consecutive photons

[2] – A number setting the threshold on the minimal multiplier of the photon rate, relative to the BG rate, above which, photons are considered part of a burst

[3] – The minimal amount of photons in a burst (its minimal size)

[4] – The mean background (BG) rate in the donor detection channel, as was retrieved by from averaging over exponential function fitting the slow process in the inter-photon time histogram, for each 20 second period of the measurement. Errors were calculated as the standard error of all 20 second BG rates.

[5] The mean background (BG) rate in the acceptor detection channel, as was retrieved by from averaging over exponential function fitting the slow process in the inter-photon time histogram, for each 20 second period of the measurement. Errors were calculated as the standard error of all 20 second BG rates.

[6] – The best fit value of the mean FRET efficiency of the 1st population out of two (the simulated mean FRET efficiency was 0.75). The reported error values are the fitting error values.

[7] - The best fit value of the FRET efficiency standard deviation of the 1st population out of two. The reported error values are the fitting error values.

[8] – The best fit value of the mean FRET efficiency of the 2nd population out of two (the simulated mean FRET efficiency was 0.50). The reported error values are the fitting error values.

[9] - The best fit value of the FRET efficiency standard deviation of the 2nd population out of two. The reported error values are the fitting error values.

[10] – The nomenclature of the sample names refers to the positions of dye labeling a dsDNA with a sequence of the lacCONS promoter. (-X)TA-(+Y)NTD – an acceptor on the base X positions upstream to the transcription start site of the promoter, on the template strand, and a donor on the base Y positions downstream to the transcription start site of the promoter , on the nontemplate strand.

[11] – no values for this population, for fits to a single gaussian model

[12] – measurement of a mixture of both FRET constructs (-8)TA-(+4)NTD & (-15)TA-(+2)NTD
