# Supplementary material for: Systematic Assessment of Burst Impurity in Confocal-Based Single-Molecule Fluorescence Detection Using Brownian Motion Simulations
Source: Molecules. 2019 Jul 13;24(14):2557. doi: 10.3390/molecules24142557 (PMC6680824; doi:10.3390/molecules24142557)
Supplement: Supplementary file 1 [file molecules-24-02557-s001.zip › SI.pdf]

Supplementary Materials

Systematic assessment of burst impurity in confocal-based single-molecule fluorescence detection using Brownian motion simulations

Dolev Hagai [1] and Eitan Lerner [1,*]

[1] Department of Biological Chemistry, The Alexander Silberman Institute of Life Sciences, Faculty of Mathematics & Science, The Edmond J. Safra Campus, The Hebrew University of Jerusalem, Jerusalem, Israel; Eitan.Lerner@mail.huji.ac.il
* Correspondence: Eitan.Lerner@mail.huji.ac.il; Tel.: +972-2-658-5457

Received: date; Accepted: date; Published: date

1.1. Reasons for bias in parameters retrieved from fluorescence autocorrelation analysis of photons in SMFD

It is worth mentioning that fluorescence autocorrelation functions at the single molecule regime (at concentrations <100 pM), where most of the time no molecule crosses the effective excitation volume, might be inaccurate. Fluorescence correlation spectroscopy (FCS) assumes the signal has a well-defined mean, and that the information is found in the temporal fluctuations about that mean. However, at the single-molecule level, there is not one mean signal, but two, one of the BG process, and the other of the fluorescence process, that occurs only once in a while. As a result, Poisson statistics do not characterize it well, but rather a combination of two Poisson processes.

When focusing on photons of bursts, however, the signal should be analyzable by FCS approaches. That might be true as long as the shape of the part of the PSF from which photons are emitted is known and resembles the Gaussian approximation. The model used for analysis of fluorescence autocorrelation functions stems from a perfectly-shaped Gaussian PSF. We, however, have already shown that after burst analysis, the molecular positions form a shape that deviates from the Gaussian shape, even when performing the simulations using a Gaussian PSF model. Therefore, the estimation of the mean amount of molecules in the effective detection volume (EDV) at any given moment, <N>, from such fits to autocorrelation curves of burst photon timestamps, may introduce biased results. Additionally, using Poisson statistics to infer the probability of more than a single molecule, P(N>1), may carry with it an additional bias, because the statistics is not pure single Poisson anymore. The statistics may get closer to Poisson statistics when using high photon rate thresholds, relative to the BG rate. Finally, the process of retrieving P(N>1) involves so many steps that propagate errors, while the information is known from the ground truth of the simulation.

Figure Legends

Figure S1. The positions of diffusing molecules (in simulations using the gaussian PSF model) when they emitted photons that were detected and selected by the burst analysis, either with minimal burst analysis parameter values (m=5 & F=6; left panels) or with stringent burst analysis parameter values (m=10, F=6 & burst size threshold, sz=40; right panels). In the top, central & bottom panels we show the 2D projections at the yz, xz & xy planes, respectively. Each dot in the scatter plots is an emitted photon. These results are for the simulation of molecules in a concentration of 62 pM, where the diffusion coefficient of the molecules was 90 μm2/s. The colors of the points correspond to the burst number out of the overall number of bursts. In each panel, the 1D projections are also shown as histograms. The black, brown and yellow contour lines align the position of the gaussian PSF model (see shapes of PSF models in Figure S1).

Figure S2. histograms of the 1D projections shown in Figures 1 & 2 for the z , y & x coordinates (left, center & right panels, respectively). From top to bottom, we assessed these histograms as a function of a sliding window of *m* consecutive photons (*m*=5, 20, 15 & 20 in blue, orange, green & red, respectively), using a constant instantaneous photon rate threshold of *F*=6; as a function of the instantaneous photon rate threshold *F*, (*F*=3, 6 ,11 ,16 & 21 in blue, orange, green, red & magenta, respectively), using a sliding window of constant *m*=10 consecutive photons; as a function of the minimal burst size threshold (10, 20, 40 & 80 in blue, orange, green & red, respectively); and as a function of the minimal burst width threshold (0.0, 0.5 & 1.0 ms in blue, orange & green, respectivey), for a constant *m*=10 & *F*=6. These results are for the simulation of molecules in a concentration of 62 pM, where the diffusion coefficient of the molecules was 90 $\mu m^2/s$. The colors of the points correspond to the burst number out of the overall number of bursts.

Figure S3. The molecular position dispersion as a function of burst search criteria and experimental conditions. Shown are the standard deviation of molecular positions in the z (left) & x (right) coordinates (the values in the y coordinate are the same as the ones in the x coordinate, in within the error ranges), when they emitted photons that were detected and selected by the burst analysis. The error values were calculated as the uncertainty of the standard deviation. All values are reported in Table S1. The assessment of the molecular position dispersion here is shown as a function of different concentrations for molecules diffusing with a constant diffusion coefficient of 90 $\mu m^2/s$, and in simulations using the gaussian PSF model.

Figure S4. The molecular position dispersion as a function of burst search criteria and experimental conditions. Shown are the standard deviation of molecular positions in the z (left) & x (right) coordinates (the values in the y coordinate are the same as the ones in the x coordinate, in within the error ranges), when they emitted photons that were detected and selected by the burst analysis. The error values were calculated as the uncertainty of the standard deviation. All values are reported in Table S1. The assessment of the molecular position dispersion here is shown as a function of molecules diffusing with different diffusion coefficients, at a constant concentration (62 pM), and in simulations using the gaussian PSF model.

Figure S5. Quantification of the level of impurity of single-molecule bursts using different burst analysis parameter values. Each panel shows a histogram of all the bursts' level of impurity, calculated as the fraction of photons arising from molecules other than the main one. The burst impurity histograms are of the simulation results after burst search analysis using a constant photon rate threshold *F*=6 and varying value of *m*, for a sliding

window of *m* consecutive photons (left), constant *m*=10 and varying *F* values (center) and constant *m*=10, *F*=6 and varying burst size threshold values (right). The continuous and dashed vertical grey lines indicate the mean and error range (as calculated using the mean and the standard error) impurity value for all bursts. These results are for the simulation of molecules in a concentration of 62 pM, where the diffusion coefficient of the molecules was 90 $\mu m^2/s$.

Figure S6. The occurrence and level of impure bursts as a function of burst search criteria and concentrations – numerical PSF model. Different burst analysis parameter values for different concentrations of molecules. The relative occurrence of impure bursts (left) was calculated as the fraction of bursts with an impurity level larger than 0 (error ranges calculated as the 95% confidence intervals), as the fraction of non-single-molecule bursts, and hence as the fraction of impure bursts. The level of impurity (right) was calculated as either the mean of all burst impurity levels (black; error ranges calculated as the standard error) or as the fraction of impure photons from all bursts relative to all burst photons (red; no error ranges, as the calculation was performed over all photons). The assessment is shown as a function of different diffusion coefficients at a constant concentration of 62 pM, and in simulations using the numerical PSF model.

Figure S7. The occurrence and level of impure bursts as a function of burst search criteria and concentrations – gaussian PSF model. Different burst analysis parameter values for different concentrations of molecules. The relative occurrence of impure bursts (left) was calculated as the fraction of bursts with an impurity level larger than 0 (error ranges calculated as the 95% confidence intervals), as the fraction of non-single-molecule bursts, and hence as the fraction of impure bursts. The level of impurity (right) was calculated as either the mean of all burst impurity levels (black; error ranges calculated as the standard error) or as the fraction of impure photons from all bursts relative to all burst photons (red; no error ranges, as the calculation was performed over all photons). The assessment is shown as a function of different concentrations for molecules diffusing with a constant diffusion coefficient of 90 μm2/s, and in simulations using the gaussian PSF model.

Figure S8. The occurrence and level of impure bursts as a function of burst search criteria and concentrations – gaussian PSF model. Different burst analysis parameter values for different concentrations of molecules. The relative occurrence of impure bursts (left) was calculated as the fraction of bursts with an impurity level larger than 0 (error ranges calculated as the 95% confidence intervals), as the fraction of non-single-molecule bursts, and hence as the fraction of impure bursts. The level of impurity (right) was calculated as either the mean of all burst impurity levels (black; error ranges calculated as the standard error) or as the fraction of impure photons from all bursts relative to all burst photons (red; no error ranges, as the calculation was performed over all photons). The assessment is shown as a function of different diffusion coefficients at a constant concentration of 62 pM, and in simulations using the gaussian PSF model.

Figure S9. The molecular positions of pure & impure bursts photons, as a function of varying burst search parameter, *m* (numerical PSF model) – shape and amplitude. We the histograms of molecular positions in the z coordinate of impure photons (red), burst photons of impure bursts (yellow), of pure bursts (green) and of all bursts (black), both un-normalized (left) to assess the weight of burst impurity, and normalized (right) to assess the histogram shapes. These results refer to the simulations in concentration of 62 pM and diffusion coefficient of 90 μm2/s, using the numerical PSF model.

Figure S10. The molecular positions of pure & impure bursts photons, as a function of varying burst size threshold *sz* (numerical PSF model) – shape and amplitude. We the histograms of molecular positions in the z coordinate of impure photons (red), burst photons of impure bursts (yellow), of pure bursts (green) and of all bursts (black), both un-normalized (left) to assess the weight of burst impurity, and normalized (right) to assess the histogram shapes. These results refer to the simulations in concentration of 62 pM and diffusion coefficient of 90 μm2/s, using the numerical PSF model.

Figure S11. The molecular positions of pure & impure bursts photons, as a function of varying the instantaneous photon rate threshold *F* (Gaussian PSF model) – shape and amplitude. We the histograms of molecular positions in the z coordinate of impure photons (red), burst photons of impure bursts (yellow), of pure bursts (green) and of all bursts (black), both un-normalized (left) to assess the weight of burst impurity, and normalized (right) to assess the histogram shapes. These results refer to the simulations in concentration of 62 pM and diffusion coefficient of 90 μm2/s, using the Gaussian PSF model.

Figure S12. The molecular positions of pure & impure bursts photons, as a function of varying burst search parameter, *m* (Gaussian PSF model) – shape and amplitude. We the histograms of molecular positions in the z coordinate of impure photons (red), burst photons of impure bursts (yellow), of pure bursts (green) and of all bursts (black), both un-normalized (left) to assess the weight of burst impurity, and normalized (right) to assess the histogram shapes. These results refer to the simulations in concentration of 62 pM and diffusion coefficient of 90 μm2/s, using the Gaussian PSF model.

Figure S13. The molecular positions of pure & impure bursts photons, as a function of varying burst size threshold *sz* (Gaussian PSF model) – shape and amplitude. We the histograms of molecular positions in the z coordinate of impure photons (red), burst photons of impure bursts (yellow), of pure bursts (green) and of all bursts (black), both un-normalized (left) to assess the weight of burst impurity, and normalized (right) to assess the histogram shapes. These results refer to the simulations in concentration of 62 pM and diffusion coefficient of 90 μm2/s, using the Gaussian PSF model.

Figure S14. Photon timestamp autocorrelations and their best fit results to a model of fluorescence correlation of freely diffusing molecules. From top to bottom: autocorrelation of all photons, of burst photons with varying the burst search parameter *m*, keeping the burst search parameter *F*=6, of burst photons with varying the burst search parameter *F*, keeping the burst search parameter *m*=10, with varying burst selection parameter, burst size threshold, keeping burst search parameters *m*=10 & *F*=6, and with varying burst selection parameter, burst width threshold, keeping burst search parameters *m*=10 & *F*=6. One can observe the trend in the mean amount of molecules in the effective detection volume at any given moment, $\langle N \rangle$, as a function of burst analysis parameter values, as the inverse of the change of the autocorrelation extrapolated to 0 lag time, τ=0.

Figure S15. The mean amount of molecules in the effective detection volume at any given moment is the same in simulations of the same concentrations. The different panels show the values of $\langle N \rangle$, the mean amount of molecules in the effective detection volume at any given moment, retrieved as best fit values from fitting the burst timestamp autocorrelation functions to a model of fluorescence autocorrelation of molecules freely diffusing in 3D, in a confocal-based setup.

Figure S16. The correlation of the probability of more than a single molecule in the effective excitation volume with molecular position dispersion. Different burst analysis parameter values for different concentrations of molecules. The probability of more than a single molecule in the effective excitation volume, *P(N>1)*, (error ranges were propagated from the values of the fitting error to the <*N*> parameter, after fitting the photon timestamp autocorrelation functions to a model of fluorescence autocorrelation, as in figure S8) were compared against the molecular position dispersion in the z coordinate (error ranges calculated as the uncertainty of the standard deviation), as a function of different burst analysis parameter values (from left to right: varying *m* values, varying *F* values, varying burst size threshold values & varying burst width threshold values), for different simulation conditions (from top to bottom: different concentrations at a constant diffusion coefficient value of 90 $\mu m^2/s$ in simulations using the gaussian PSF model, different concentrations at a constant diffusion coefficient value of 90 $\mu m^2/s$ in simulations using the numerical PSF model, different diffusion coefficients at a constant concentration of 62 pM in simulations using the gaussian PSF model, and , different diffusion coefficients at a constant concentration of 62 pM in simulations using the numerical PSF model).

Figure S17. Two estimates of the diffusion time through the effective detection volume, and their usefulness. Shown are the values of the mean of all burst widths (error calculated as standard error), the mean diffusion time as was retrieved from best fits of fluorescence autocorrelation model of freely diffusing molecules in a confocal-setup, to the photon timestamp autocorrelation functions, as in figure S8 (errors are fitting errors). The figure shows the minimal error ranges of burst widths, the large error values of the diffusion times, and the lack of correlation between the mean values, rendering the diffusion time values useless for accurate time estimates, and the mean burst durations useful time estimates, when the simulation used either gaussian or numerical PSF models (left or right, respectively). Top to bottom: varying the burst search parameter *m* and keeping the burst search parameter *F*=6, varying the burst search parameter *F* and keeping the burst search parameter *m*=10, varying burst size threshold and keeping burst search parameters *m*=10 & *F*=6, and varying burst width threshold and keeping burst search parameters *m*=10 & *F*=6.

Figure S18. Estimating mean burst widths. Shown are the histograms of all burst widths after testing burst analysis results with (from top to bottom) varying values of the burst search parameter *m* and a constant instantaneous photon rate threshold *F*=6, varying values of the instantaneous photon rate threshold F and a constant burst search parameter value of *m*=10, varying values of the burst size threshold *sz* and constant burst search parameter values *m*=10 & *F*=6, and varying values of the burst width threshold *w* and constant burst search parameter values *m*=10 & *F*=6. Continuous vertical lines indicate the mean burst widths and dashed vertical lines indicate the error ranges, calculated from the mean and the standard error of the burst widths. These results refer to the simulations in concentration of 62 pM and diffusion coefficient of 90 μm2/s, using the numerical PSF model.

Figure S19. The correlation of the mean burst widths with the molecular position dispersion. The mean burst widths, (error ranges were calculated as standard errors) were compared against the molecular position dispersion in the z coordinate (error ranges calculated as the uncertainty of the standard deviation), as a function of different burst analysis parameter values (from left to right: varying *m* values, varying *F* values, varying burst size threshold values & varying burst width threshold values), the molecular dispersion in z and x coordinates show as a pair of panels for different simulation conditions (from top to bottom: different concentrations at a

constant diffusion coefficient value of 90 μm²/s in simulations using the gaussian PSF model, different concentrations at a constant diffusion coefficient value of 90 μm²/s in simulations using the numerical PSF model, different diffusion coefficients at a constant concentration of 62 pM in simulations using the gaussian PSF model, and , different diffusion coefficients at a constant concentration of 62 pM in simulations using the numerical PSF model).

Figure S20. Simulations of smFRET with two FRET subpopulations, with best fit results to a sum-of-two-gaussians model with a fixed fraction value f=0.6666. From top to bottom, each panel shows the resulting FRET histogram (blue), the best fit sum of two-gaussians, with a fixed population fraction value f=0.6666 (red), the best-fit mean FRET efficiencies (orange and cyan vertical lines; dimmer lines show the error ranges), and the simulation ground-truth mean FRET efficiency values (dashed red and green vertical lines). These results are for the 60 second simulation of molecules a concentration of 62 pM, where the diffusion coefficient of the molecules was 90 μm²/s, using the numerical PSF model, and the molecules were split to 10 with E=0.75 & 5 with E=0.5. The number of bursts in each histogram is also reported in each panel. The best fit values and the fitting error values are also reported in Table S2.

Figure S21. The values of the retrieved quantities are the same within the error ranges for 60 and 180 second simulations. Simulations lasting either 60 or 180 seconds were performed and then analyzed using the different burst analysis parameter values used over the whole work. The figure shows the values of the retrieved quantities for the 60 seconds (black) and 180 seconds (red) simulations, testing them either for fast or slow diffusion coefficients (90 or 5.625 μm²/s, at a constant concentration of 62 pM), and against either gaussian or numerical PSF models.

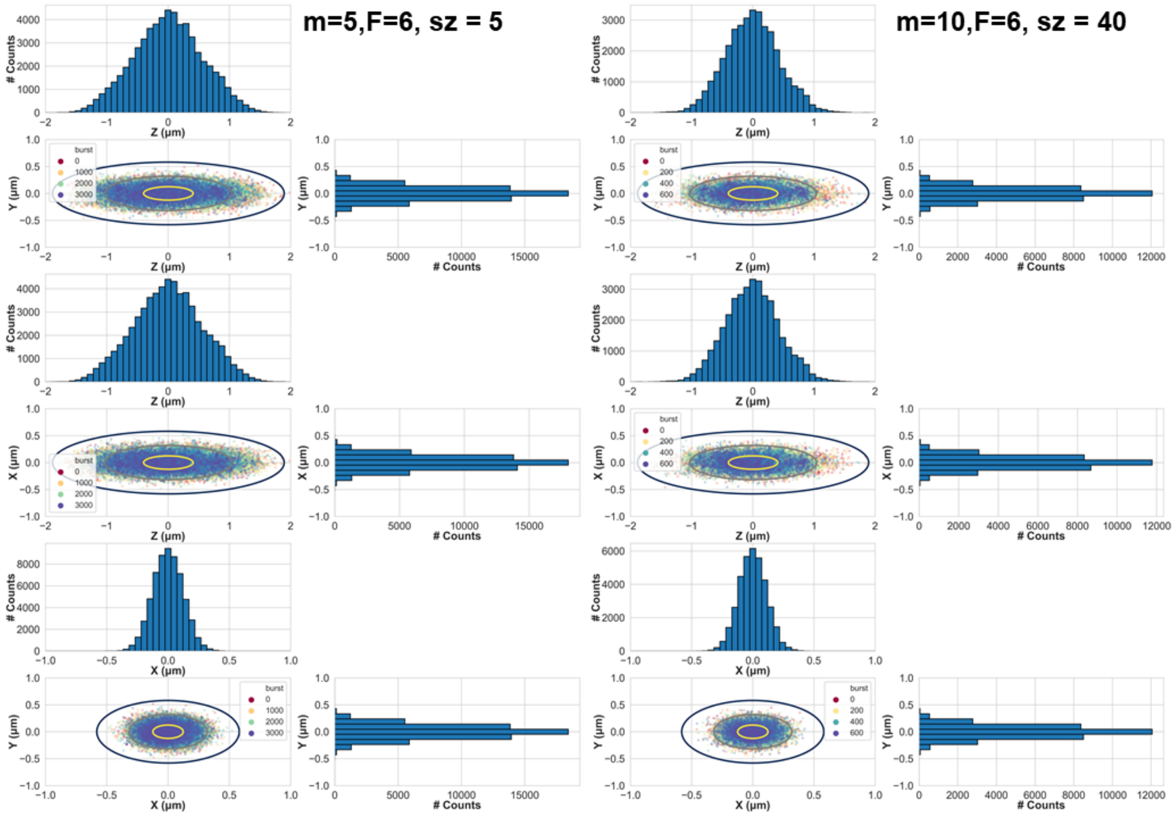
m=5,F=6, sz = 5
m=10,F=6, sz = 40
# Counts
Z (μm)
Y (μm)
X (μm)
burst
0
1000
2000
3000
200
400
600

Figure S1.

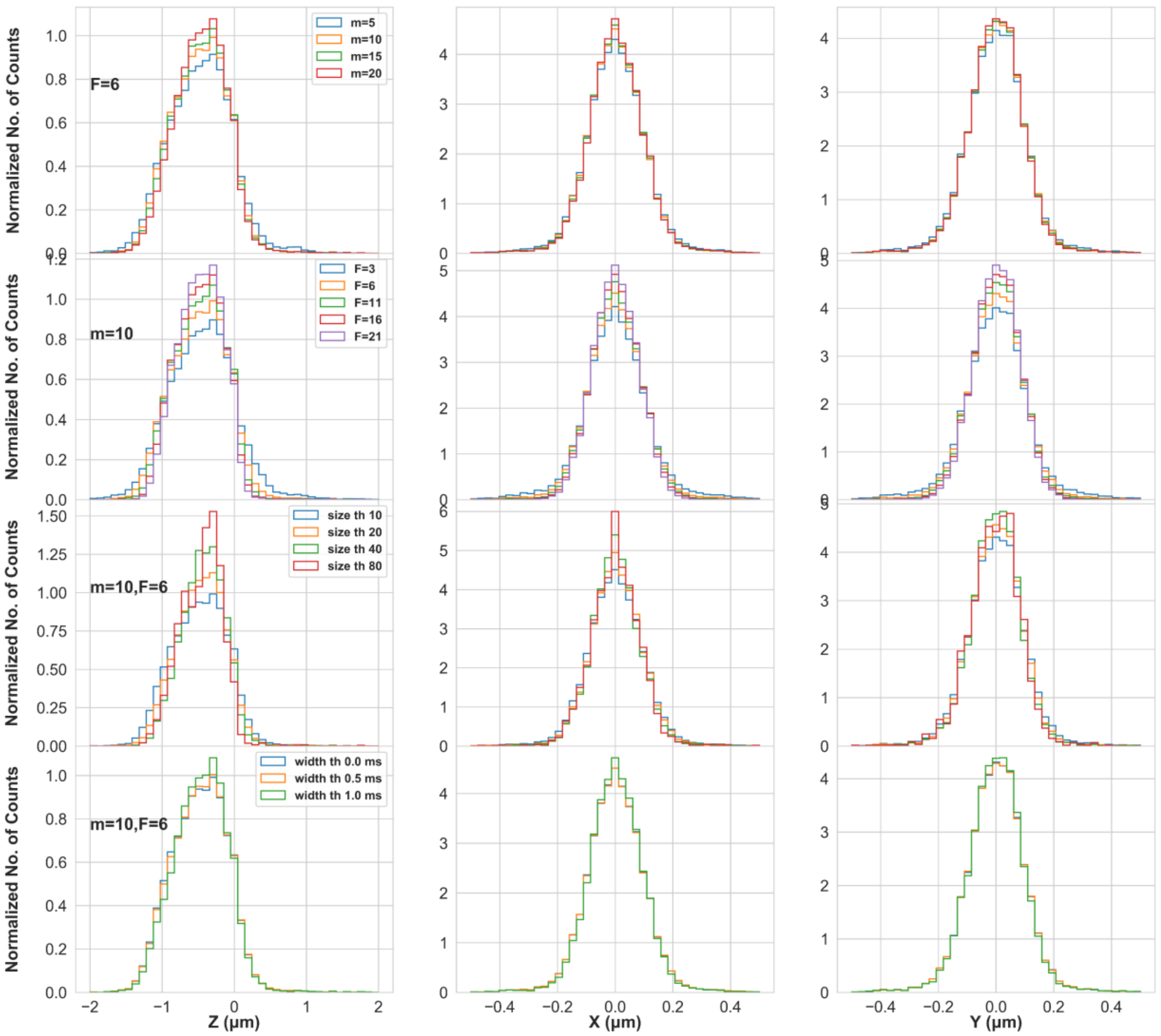
m=5
m=10
m=15
m=20
F=6
F=3
F=6
F=11
F=16
F=21
m=10
size th 10
size th 20
size th 40
size th 80
m=10,F=6
width th 0.0 ms
width th 0.5 ms
width th 1.0 ms
m=10,F=6
Normalized No. of Counts
Z (μm)
X (μm)
Y (μm)

Figure S2.

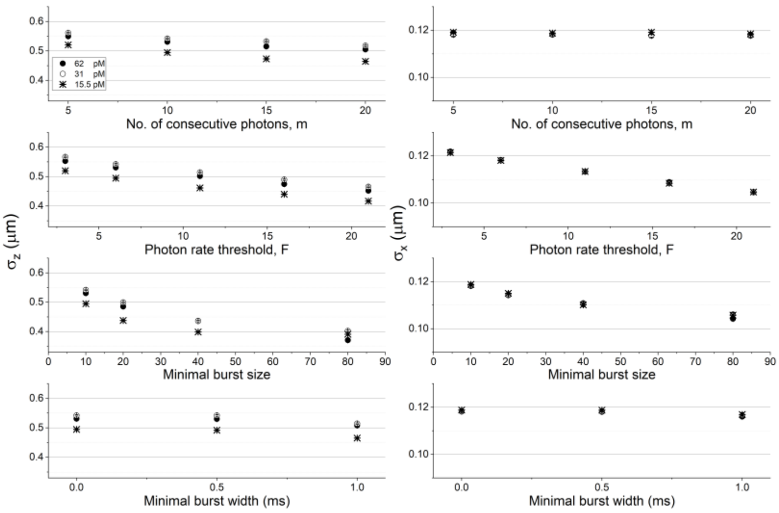
62 pM
31 pM
15.5 pM
σz (µm)
σx (µm)
No. of consecutive photons, m
Photon rate threshold, F
Minimal burst size
Minimal burst width (ms)

Figure S3

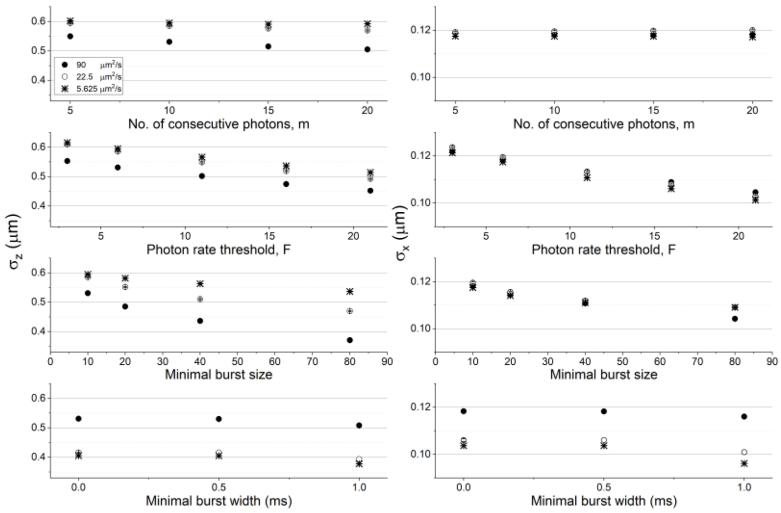
90 μm²/s
22.5 μm²/s
5.625 μm²/s
No. of consecutive photons, m
Photon rate threshold, F
Minimal burst size
Minimal burst width (ms)
σz (μm)
σx (μm)

Figure S4

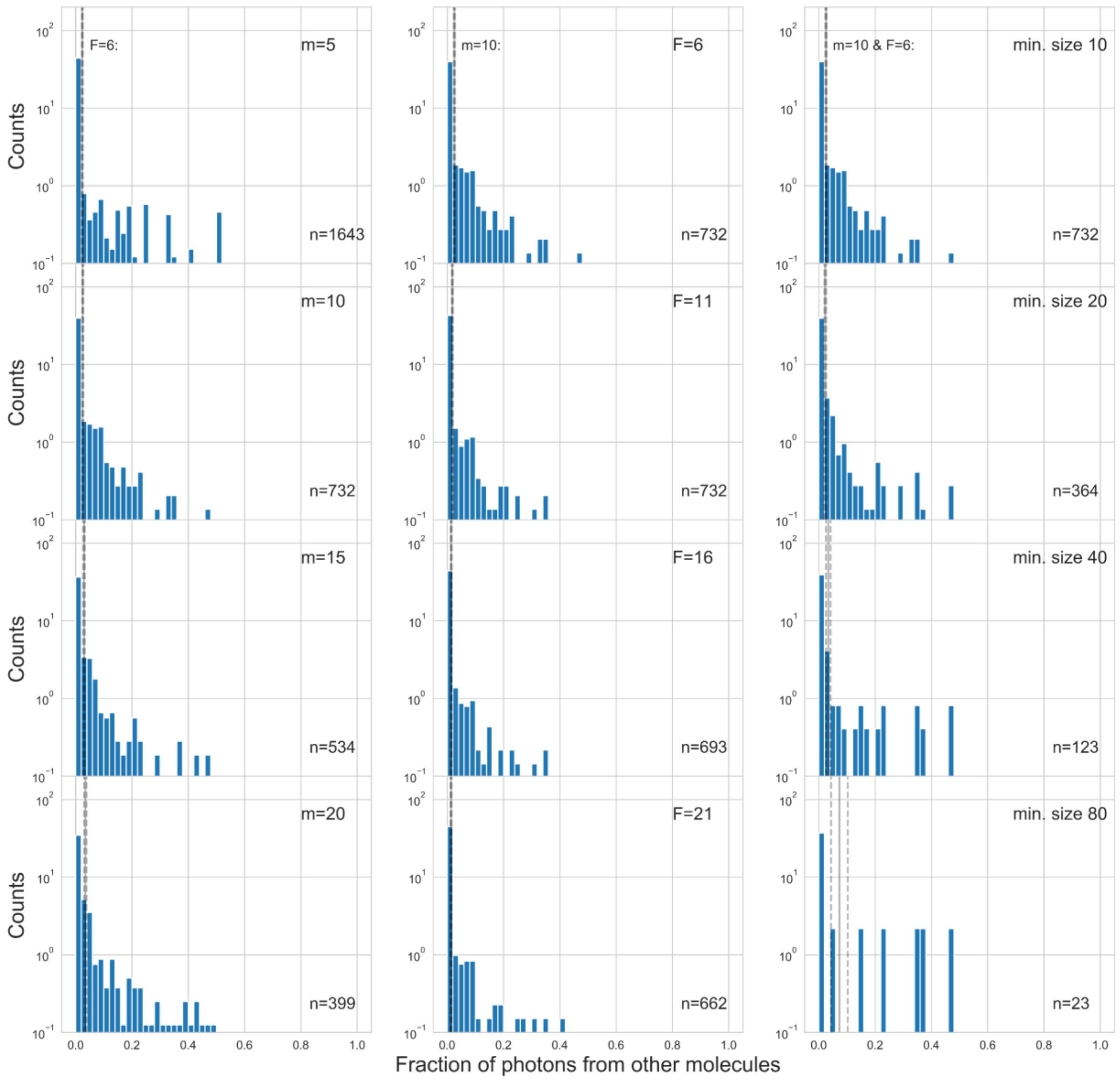
F=6:
m=5
n=1643
m=10
n=732
m=15
n=534
m=20
n=399
m=10:
F=6
n=732
F=11
n=732
F=16
n=693
F=21
n=662
m=10 & F=6:
min. size 10
n=732
min. size 20
n=364
min. size 40
n=123
min. size 80
n=23
Counts
Fraction of photons from other molecules

Figure S5

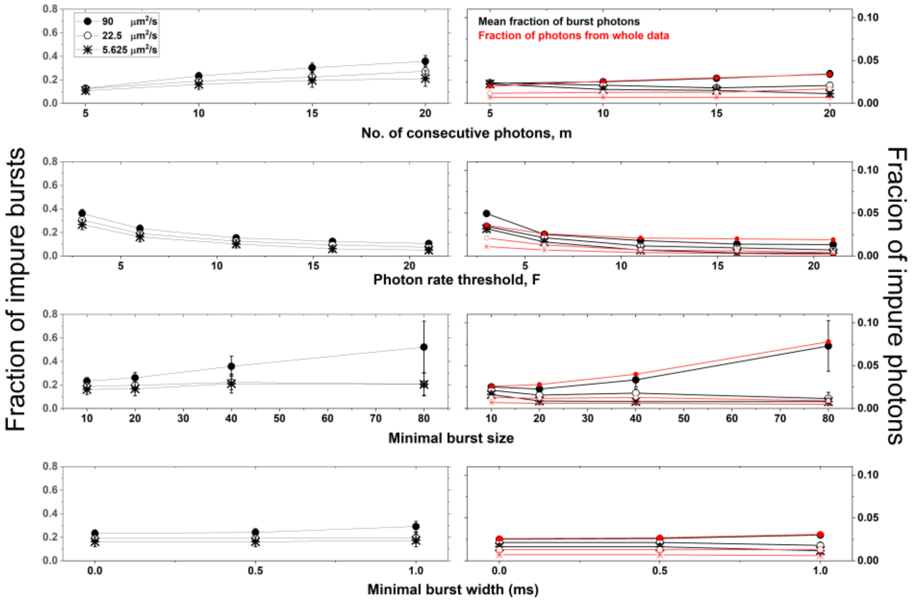
90 μm²/s
22.5 μm²/s
5.625 μm²/s
Mean fraction of burst photons
Fraction of photons from whole data
Fraction of impure bursts
Fracion of impure photons
No. of consecutive photons, m
Photon rate threshold, F
Minimal burst size
Minimal burst width (ms)

Figure S6

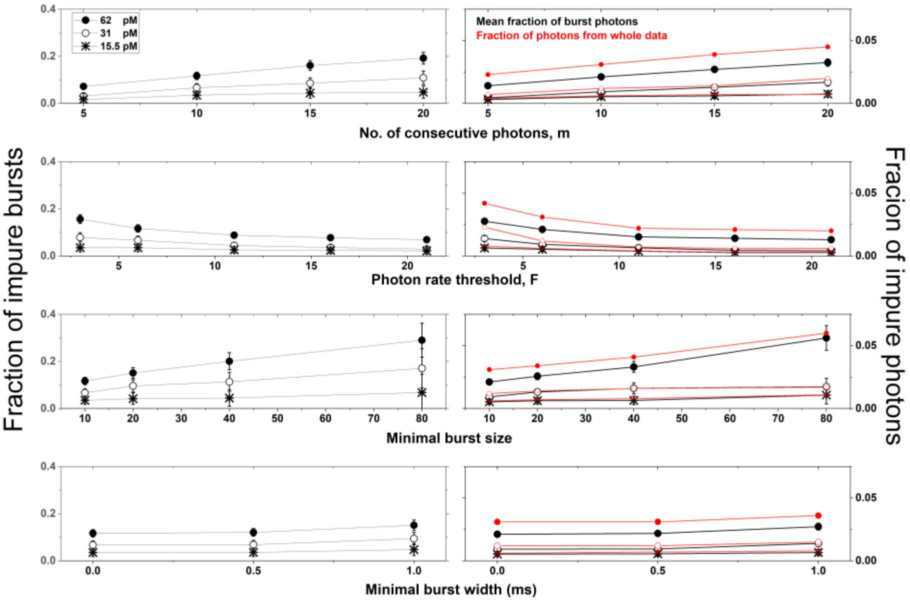
62 pM
31 pM
15.5 pM
Mean fraction of burst photons
Fraction of photons from whole data
No. of consecutive photons, m
Photon rate threshold, F
Minimal burst size
Minimal burst width (ms)
Fraction of impure bursts
Fracion of impure photons

Figure S7

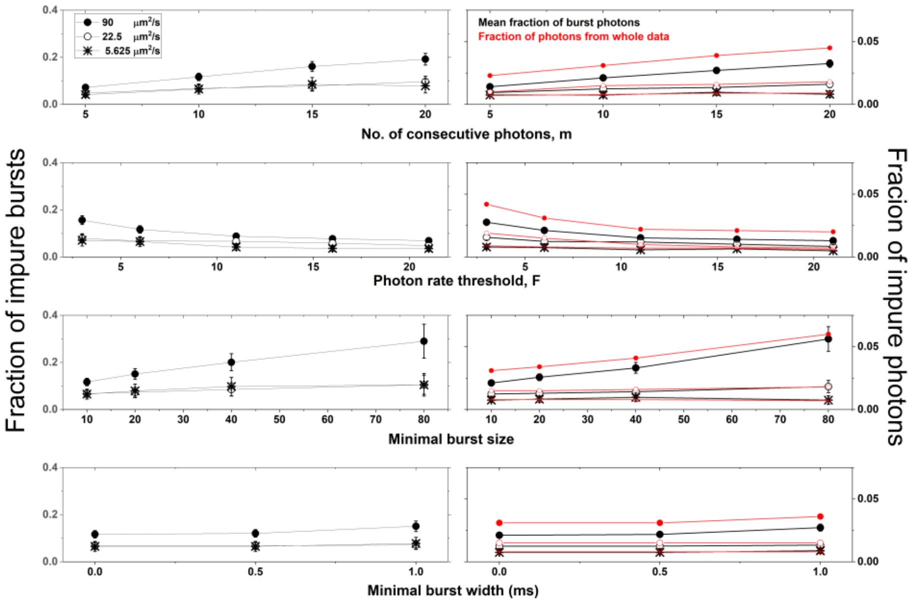
90 μm²/s
22.5 μm²/s
5.625 μm²/s
Mean fraction of burst photons
Fraction of photons from whole data
No. of consecutive photons, m
Photon rate threshold, F
Minimal burst size
Minimal burst width (ms)
Fraction of impure bursts
Fracion of impure photons

Figure S8

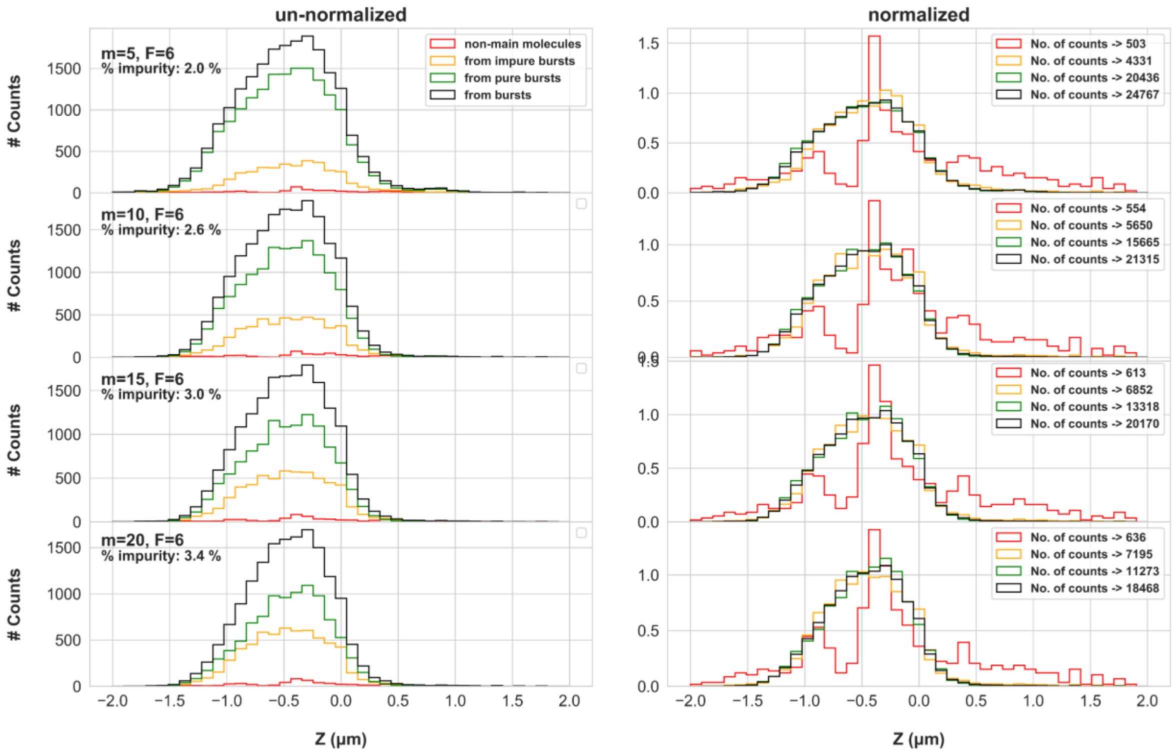
un-normalized
normalized
m=5, F=6
% impurity: 2.0 %
m=10, F=6
% impurity: 2.6 %
m=15, F=6
% impurity: 3.0 %
m=20, F=6
% impurity: 3.4 %
non-main molecules
from impure bursts
from pure bursts
from bursts
No. of counts -> 503
No. of counts -> 4331
No. of counts -> 20436
No. of counts -> 24767
No. of counts -> 554
No. of counts -> 5650
No. of counts -> 15665
No. of counts -> 21315
No. of counts -> 613
No. of counts -> 6852
No. of counts -> 13318
No. of counts -> 20170
No. of counts -> 636
No. of counts -> 7195
No. of counts -> 11273
No. of counts -> 18468
Counts
Z (μm)

Figure S9

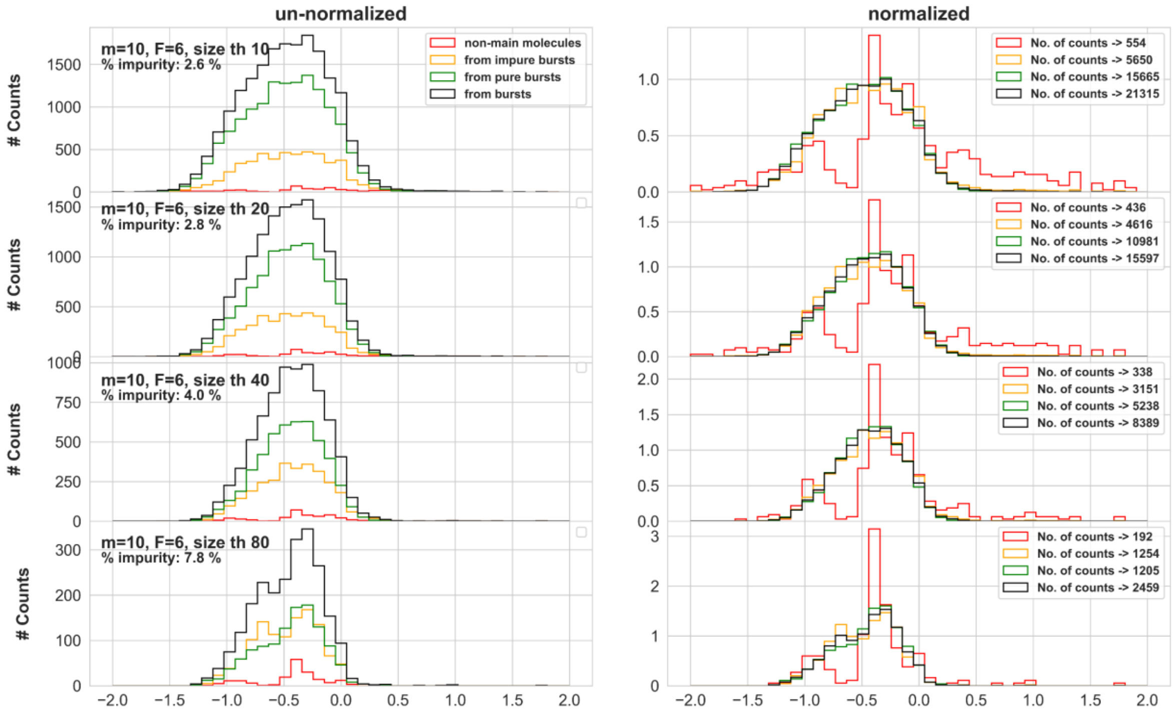
un-normalized
normalized
m=10, F=6, size th 10
% impurity: 2.6 %
m=10, F=6, size th 20
% impurity: 2.8 %
m=10, F=6, size th 40
% impurity: 4.0 %
m=10, F=6, size th 80
% impurity: 7.8 %
non-main molecules
from impure bursts
from pure bursts
from bursts
Counts
No. of counts -> 554
No. of counts -> 5650
No. of counts -> 15665
No. of counts -> 21315
No. of counts -> 436
No. of counts -> 4616
No. of counts -> 10981
No. of counts -> 15597
No. of counts -> 338
No. of counts -> 3151
No. of counts -> 5238
No. of counts -> 8389
No. of counts -> 192
No. of counts -> 1254
No. of counts -> 1205
No. of counts -> 2459

Figure S10

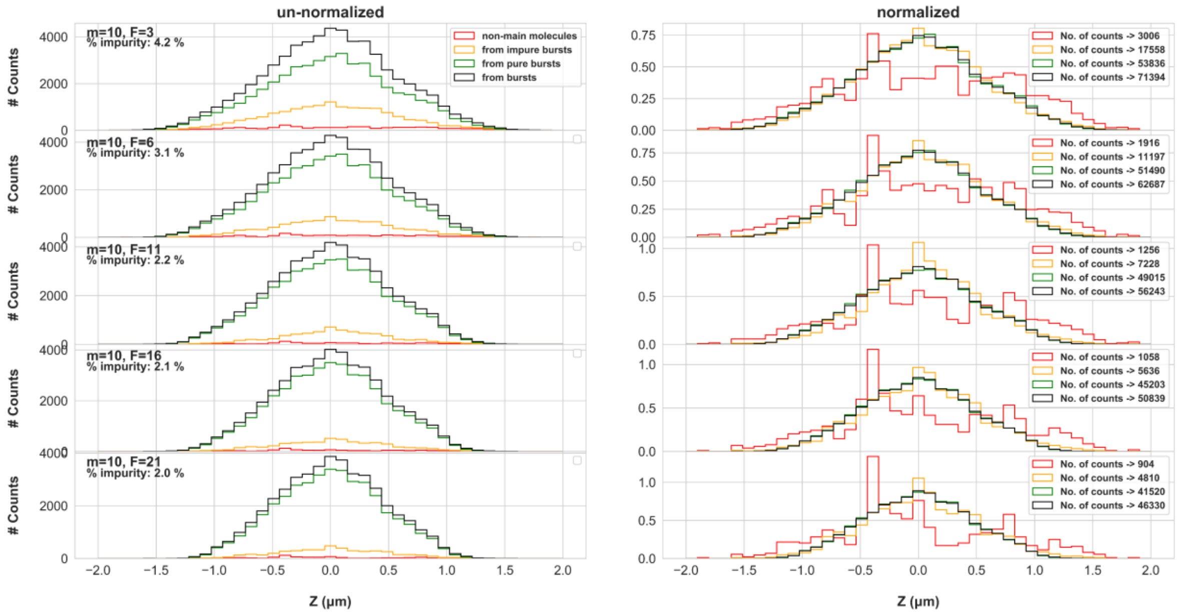

un-normalized
normalized
m=10, F=3
% impurity: 4.2 %
m=10, F=6
% impurity: 3.1 %
m=10, F=11
% impurity: 2.2 %
m=10, F=16
% impurity: 2.1 %
m=10, F=21
% impurity: 2.0 %
non-main molecules
from impure bursts
from pure bursts
from bursts
No. of counts -> 3006
No. of counts -> 17558
No. of counts -> 53836
No. of counts -> 71394
No. of counts -> 1916
No. of counts -> 11197
No. of counts -> 51490
No. of counts -> 62687
No. of counts -> 1256
No. of counts -> 7228
No. of counts -> 49015
No. of counts -> 56243
No. of counts -> 1058
No. of counts -> 5636
No. of counts -> 45203
No. of counts -> 50839
No. of counts -> 904
No. of counts -> 4810
No. of counts -> 41520
No. of counts -> 46330
Counts
Z (μm)

Figure S11

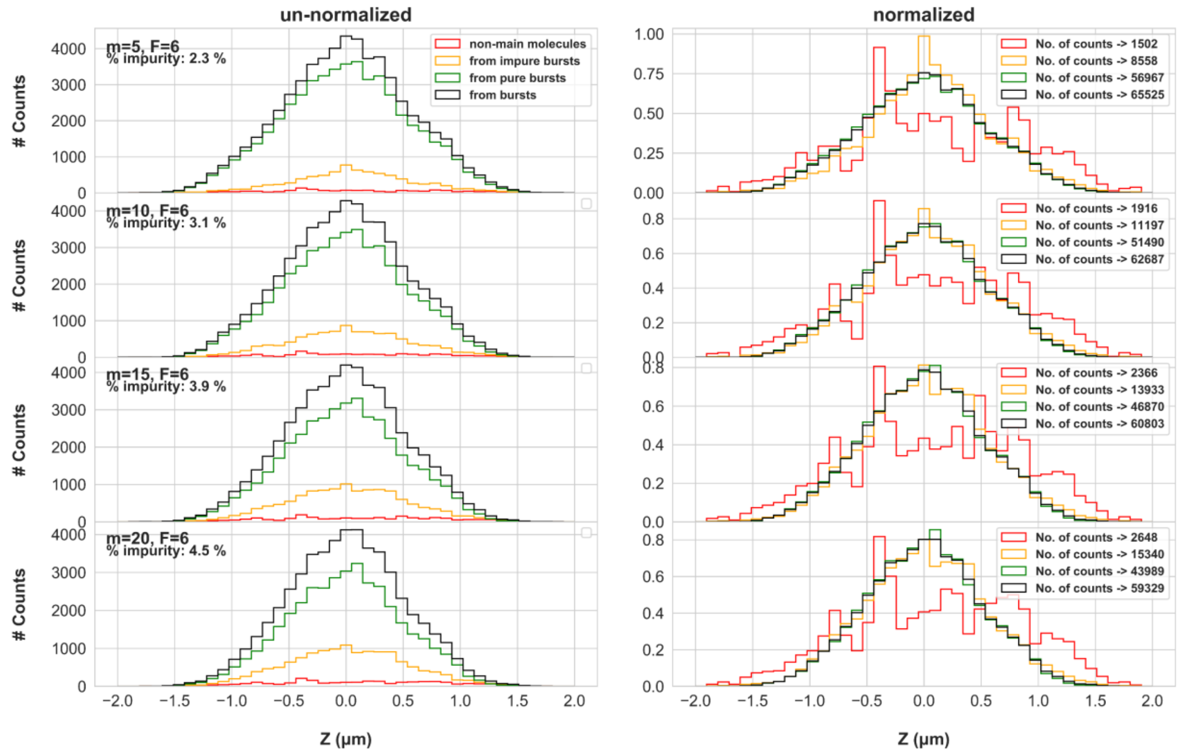
un-normalized
normalized
m=5, F=6
% impurity: 2.3 %
non-main molecules
from impure bursts
from pure bursts
from bursts
No. of counts -> 1502
No. of counts -> 8558
No. of counts -> 56967
No. of counts -> 65525
m=10, F=6
% impurity: 3.1 %
No. of counts -> 1916
No. of counts -> 11197
No. of counts -> 51490
No. of counts -> 62687
m=15, F=6
% impurity: 3.9 %
No. of counts -> 2366
No. of counts -> 13933
No. of counts -> 46870
No. of counts -> 60803
m=20, F=6
% impurity: 4.5 %
No. of counts -> 2648
No. of counts -> 15340
No. of counts -> 43989
No. of counts -> 59329
Counts
Z (μm)
Z (μm)

Figure S12

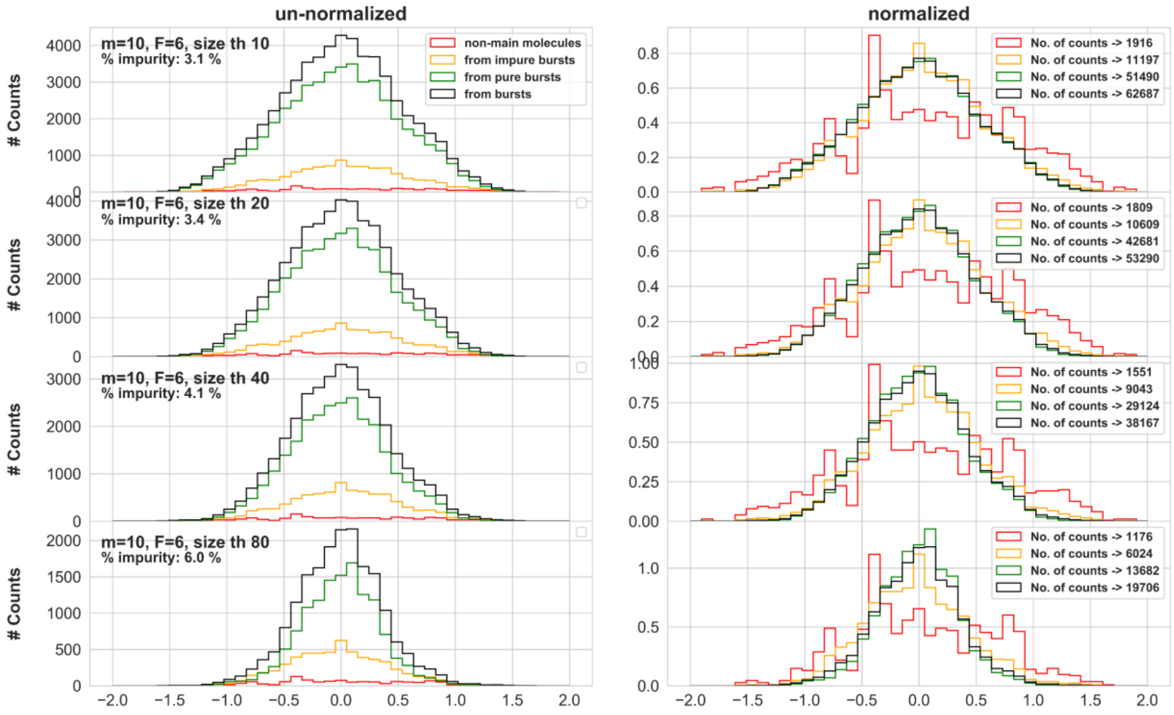

un-normalized
normalized
m=10, F=6, size th 10
% impurity: 3.1 %
non-main molecules
from impure bursts
from pure bursts
from bursts
No. of counts -> 1916
No. of counts -> 11197
No. of counts -> 51490
No. of counts -> 62687
m=10, F=6, size th 20
% impurity: 3.4 %
No. of counts -> 1809
No. of counts -> 10609
No. of counts -> 42681
No. of counts -> 53290
m=10, F=6, size th 40
% impurity: 4.1 %
No. of counts -> 1551
No. of counts -> 9043
No. of counts -> 29124
No. of counts -> 38167
m=10, F=6, size th 80
% impurity: 6.0 %
No. of counts -> 1176
No. of counts -> 6024
No. of counts -> 13682
No. of counts -> 19706
Counts

Figure S13

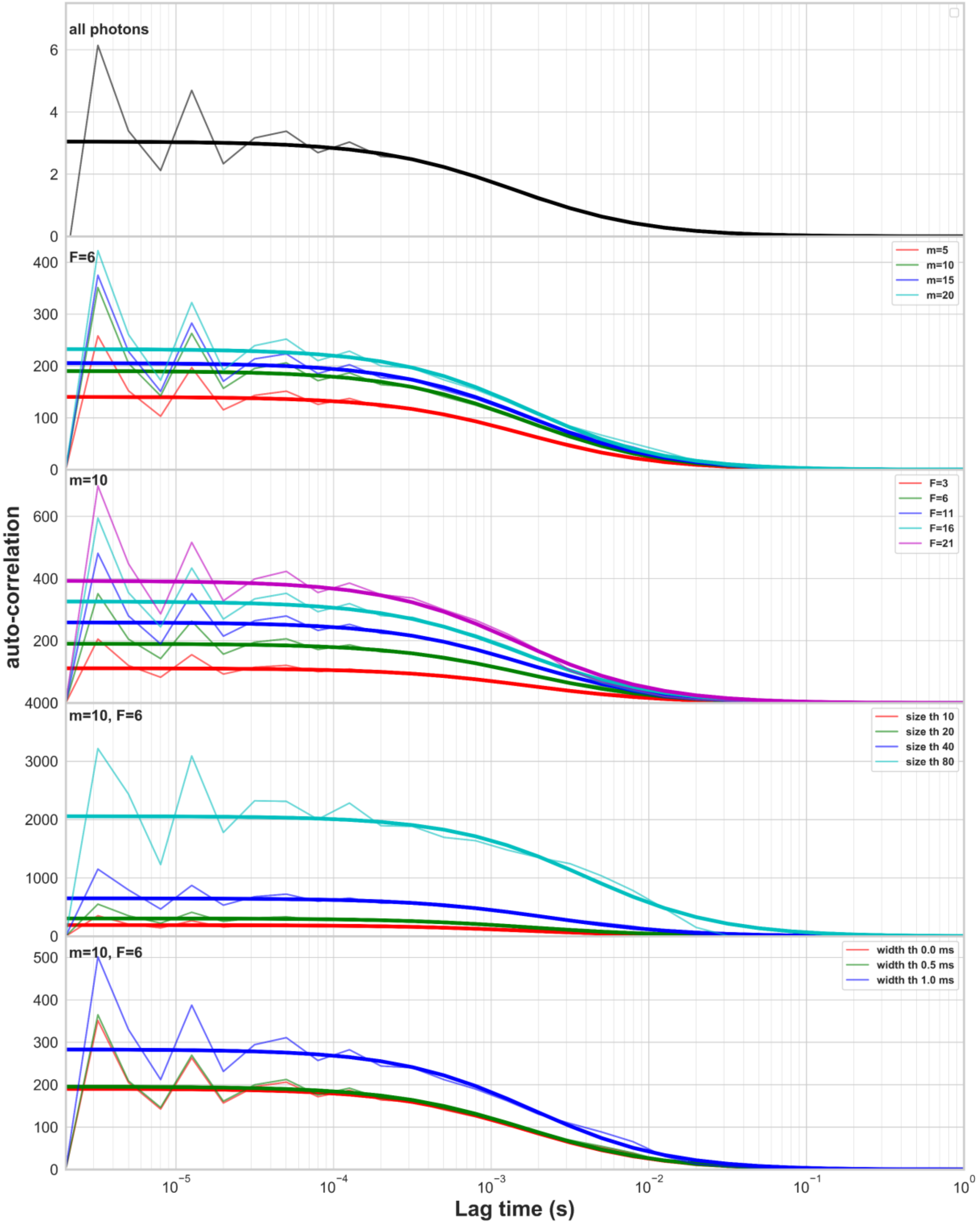
all photons
F=6
m=5
m=10
m=15
m=20
m=10
F=3
F=6
F=11
F=16
F=21
m=10, F=6
size th 10
size th 20
size th 40
size th 80
m=10, F=6
width th 0.0 ms
width th 0.5 ms
width th 1.0 ms
auto-correlation
Lag time (s)

Figure S14

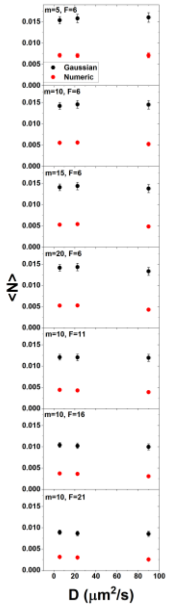
m=5, F=6
Gaussian
Numeric
m=10, F=6
m=15, F=6
m=20, F=6
m=10, F=11
m=10, F=16
m=10, F=21
0.000
0.005
0.010
0.015
<N>
0
20
40
60
80
100
D (μm²/s)

Figure S15

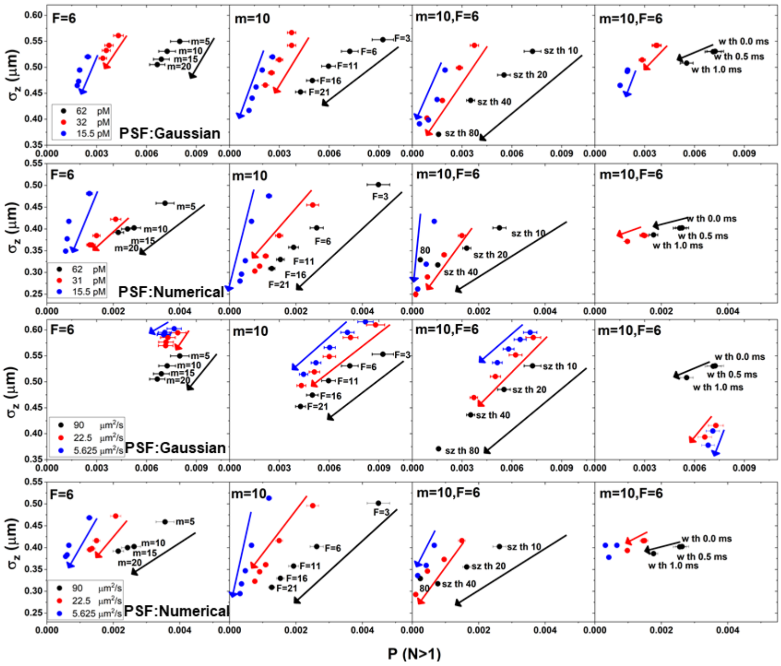
F=6
m=10
m=10,F=6
m=10,F=6
PSF:Gaussian
PSF:Numerical
62 pM
32 pM
15.5 pM
31 pM
90 µm²/s
22.5 µm²/s
5.625 µm²/s
m=5
m=10
m=15
m=20
F=3
F=6
F=11
F=16
F=21
sz th 10
sz th 20
sz th 40
sz th 80
w th 0.0 ms
w th 0.5 ms
w th 1.0 ms
σz (µm)
P (N>1)

Figure S16

293

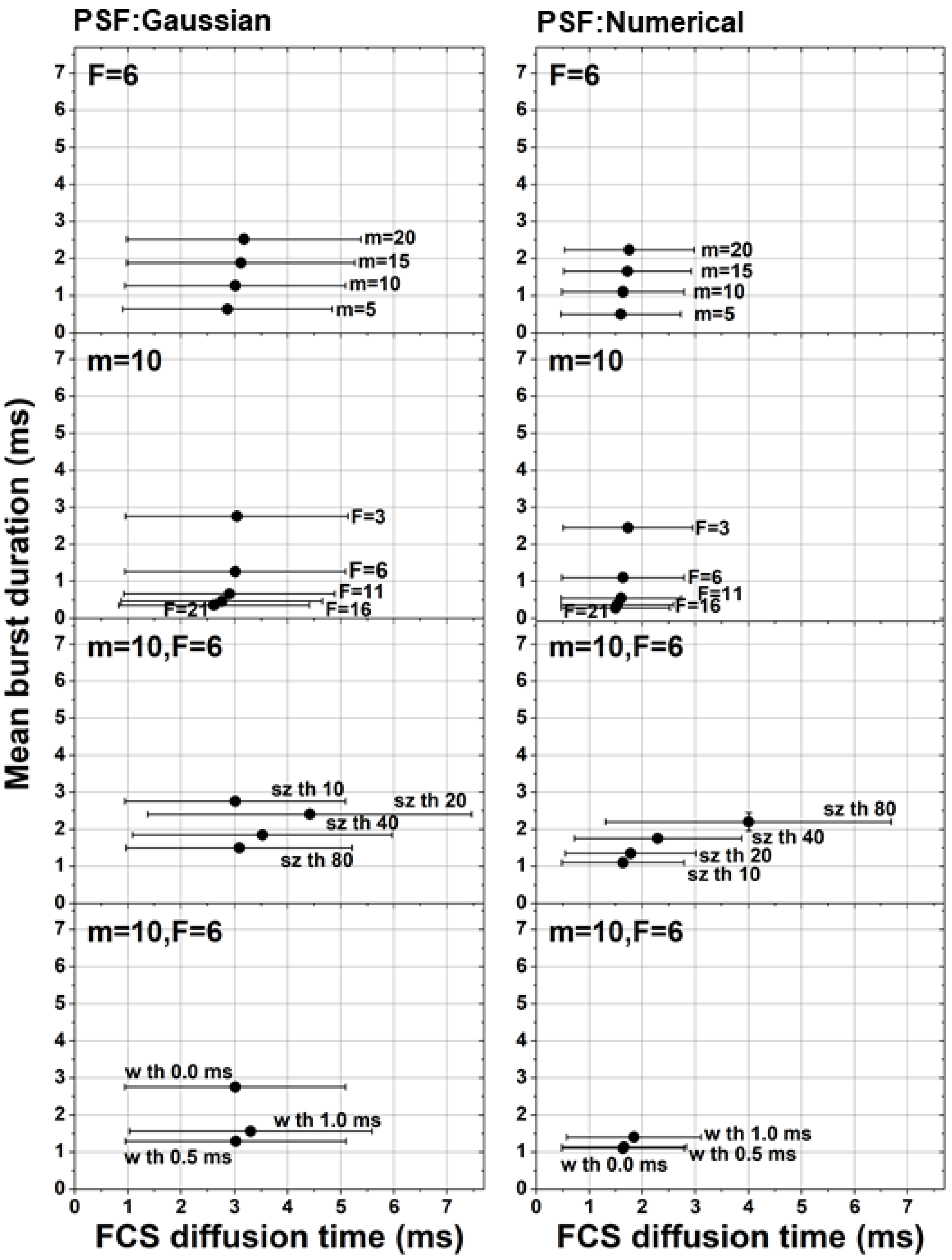

294

295 **Figure S17**

296

297

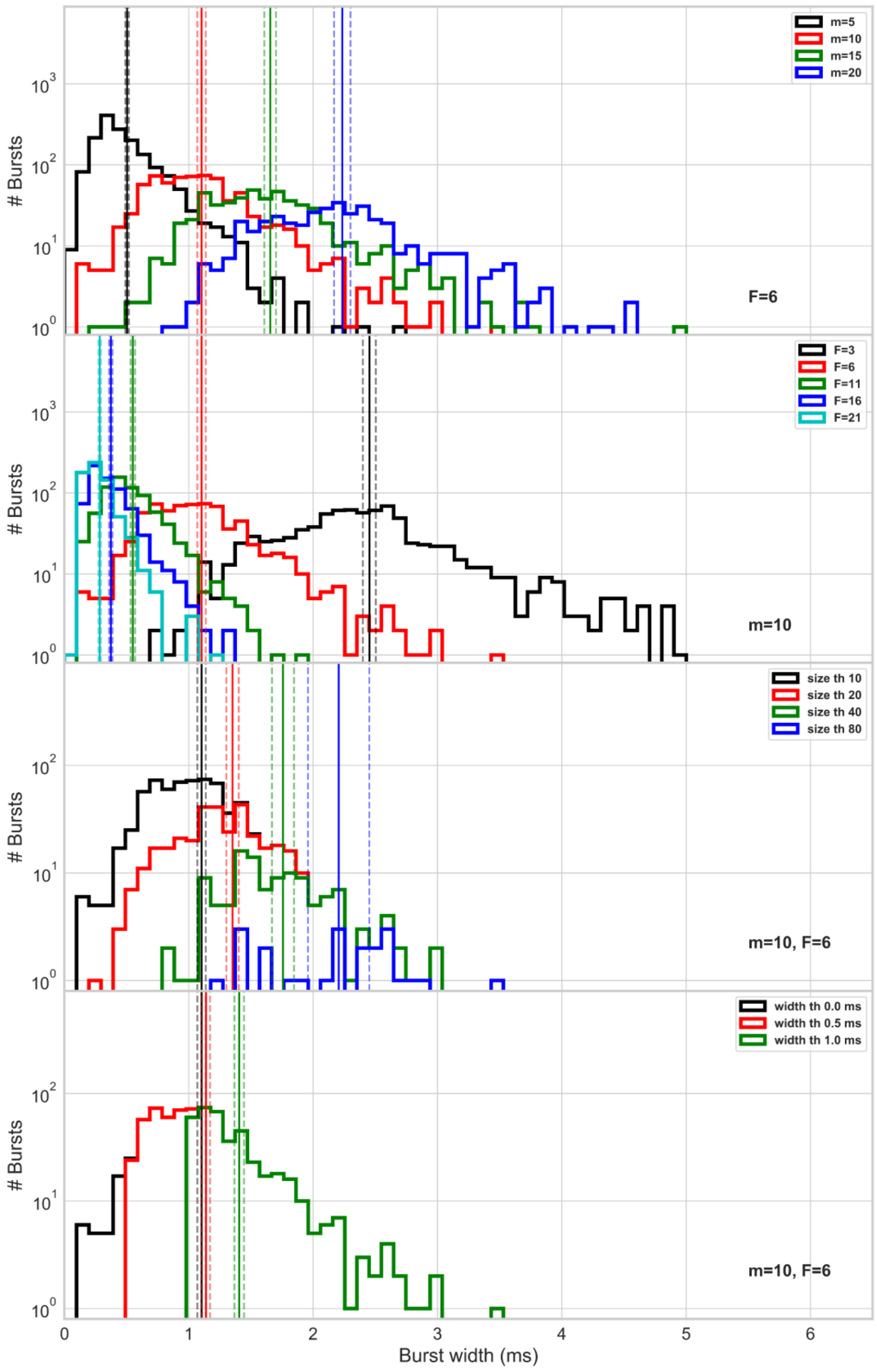

298

299 **Figure S18**

300

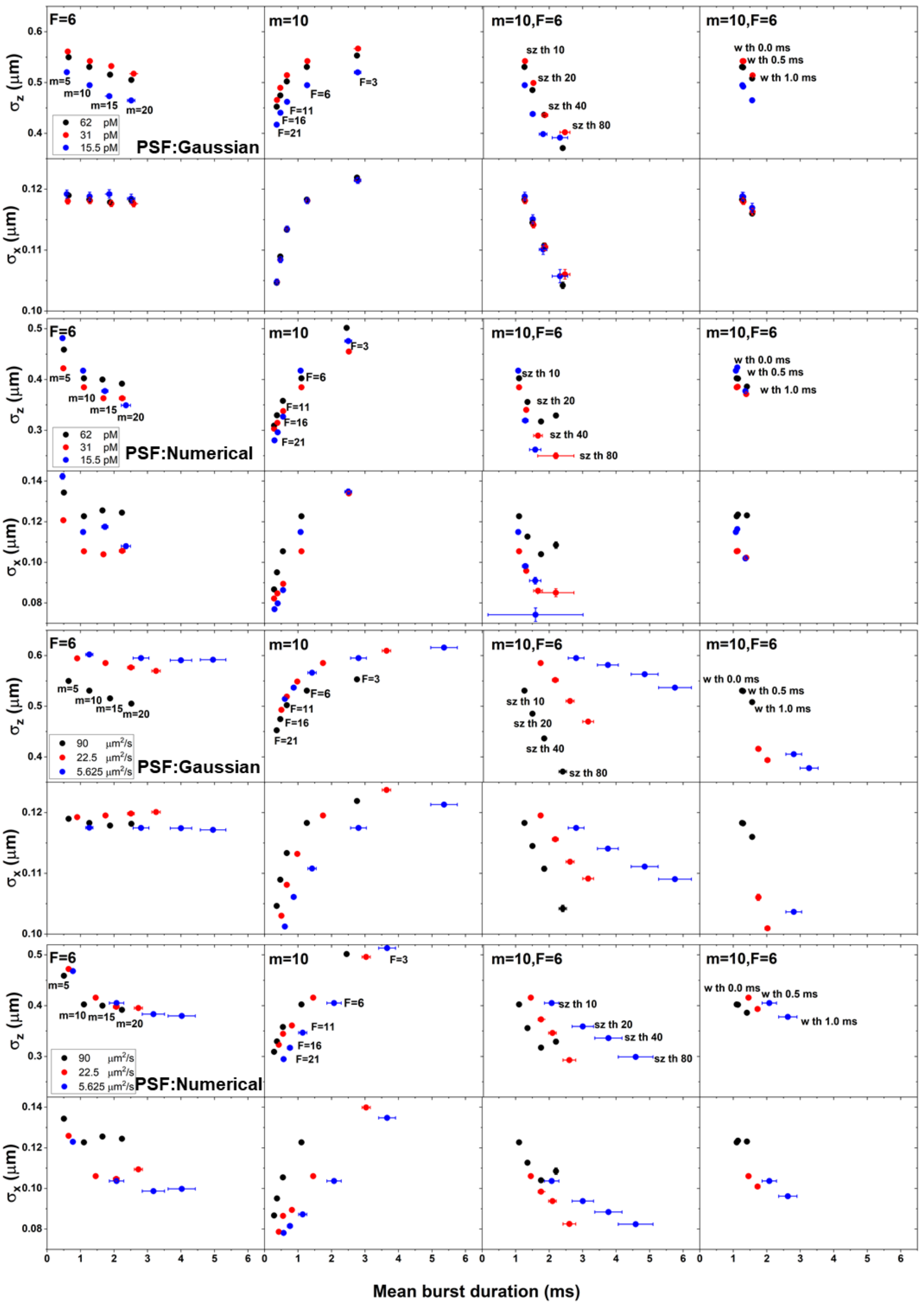
F=6
m=10
m=10,F=6
σz (µm)
σx (µm)
m=5
m=10
m=15
m=20
62 pM
31 pM
15.5 pM
PSF:Gaussian
PSF:Numerical
F=3
F=6
F=11
F=16
F=21
sz th 10
sz th 20
sz th 40
sz th 80
w th 0.0 ms
w th 0.5 ms
w th 1.0 ms
90 µm²/s
22.5 µm²/s
5.625 µm²/s
Mean burst duration (ms)

Figure S19

305

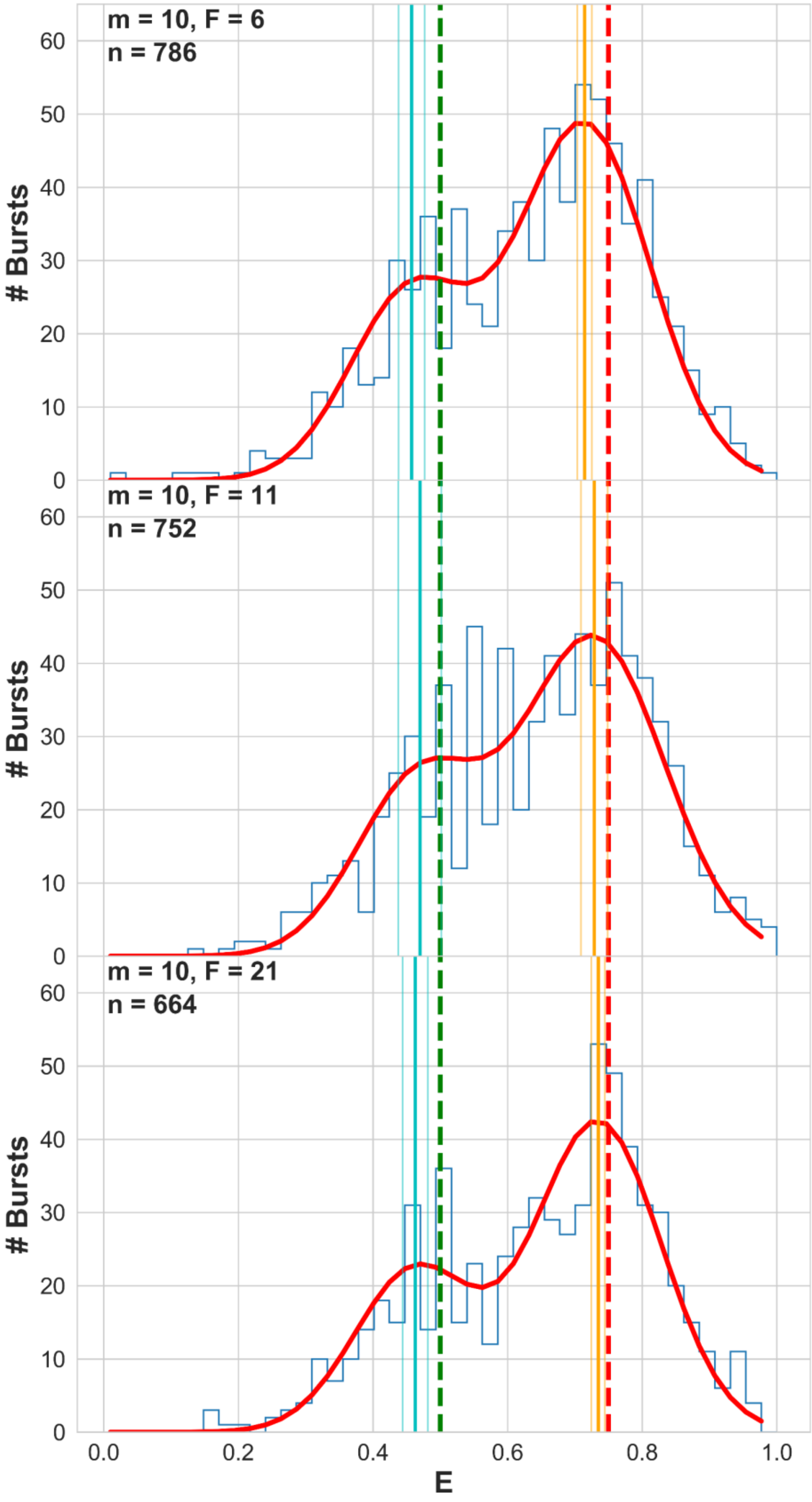

306

307 **Figure S20**

308

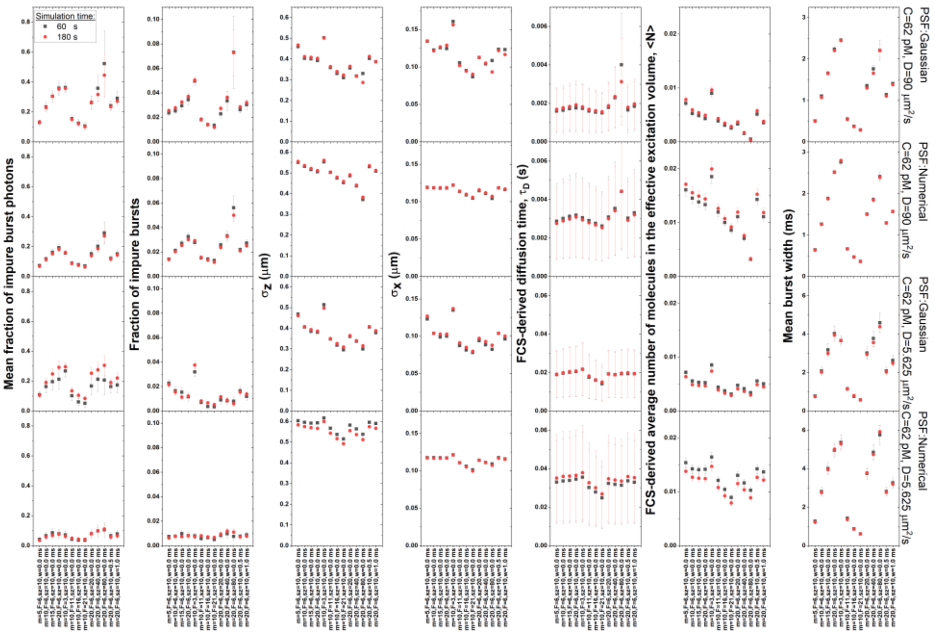
Simulation time:
60 s
180 s
Mean fraction of impure burst photons
Fraction of impure bursts
FCS-derived diffusion time, τ_D (s)
FCS-derived average number of molecules in the effective excitation volume, <N>
Mean burst width (ms)
PSF:Gaussian C=62 pM, D=90 μm²/s
PSF:Numerical C=62 pM, D=90 μm²/s
PSF:Gaussian C=62 pM, D=5.625 μm²/s
PSF:Numerical C=62 pM, D=5.625 μm²/s

Figure S21

molecules

MDPI

312
